# Oxidative Stress Differentially Influences the Survival and Metabolism of Cells in the Melanoma Microenvironment

**DOI:** 10.3390/cells11060930

**Published:** 2022-03-08

**Authors:** Emily R. Trzeciak, Niklas Zimmer, Isabelle Gehringer, Lara Stein, Barbara Graefen, Jonathan Schupp, Achim Stephan, Stephan Rietz, Michael Prantner, Andrea Tuettenberg

**Affiliations:** 1 Department of Dermatology, University Medical Center Mainz, Johannes Gutenberg University Mainz, 55131 Mainz, Germany; etrzecia@students.uni-mainz.de (E.R.T.); niklas.zimmer@unimedizin-mainz.de (N.Z.); gehringer.isabelle@gmail.com (I.G.); larstein@uni-mainz.de (L.S.); bagraefe@uni-mainz.de (B.G.); jonathan.schupp@kgu.de (J.S.); stephan.rietz@unimedizin-mainz.de (S.R.); 2 Institute of Immunology, University Medical Center Mainz, Johannes Gutenberg University Mainz, 55131 Mainz, Germany; 3 Institute of Neurology (Edinger Institute), Goethe University Hospital Frankfurt, 60528 Frankfurt, Germany; 4 Frankfurt Cancer Institute, 60596 Frankfurt, Germany; 5 BOWA-Electronic GmbH & Co. KG, 72810 Gomaringen, Germany; achim.stephan@bowa-medical.com (A.S.); michael.prantner@bowa-medical.com (M.P.); 6 Research Center for Immunotherapy, University Medical Center Mainz, Johannes Gutenberg University Mainz, 55131 Mainz, Germany

**Keywords:** tumor microenvironment, metabolic alterations, tumor therapy, cold atmospheric plasma, tumor spheroids, plasma jet, malignant melanoma, T cell, macrophage

## Abstract

The cellular composition of the tumor microenvironment, including tumor, immune, stromal, and endothelial cells, significantly influences responses to cancer therapies. In this study, we analyzed the impact of oxidative stress, induced by cold atmospheric plasma (CAP), on tumor cells, T cells, and macrophages, which comprise part of the melanoma microenvironment. To accomplish this, cells were grown in different in vitro cell culture models and were treated with varying amounts of CAP. Subsequent alterations in viability, proliferation, and phenotype were analyzed via flow cytometry and metabolic alterations by Seahorse Cell Mito Stress Tests. It was found that cells generally exhibited reduced viability and proliferation, stemming from CAP induced G2/M cell cycle arrest and subsequent apoptosis, as well as increased mitochondrial stress following CAP treatment. Overall, sensitivity to CAP treatment was found to be cell type dependent with T cells being the most affected. Interestingly, CAP influenced the polarization of M0 macrophages to a “M0/M2-like” phenotype, and M1 macrophages were found to display a heightened sensitivity to CAP induced mitochondrial stress. CAP also inhibited the growth and killed melanoma cells in 2D and 3D in vitro cell culture models in a dose-dependent manner. Improving our understanding of oxidative stress, mechanisms to manipulate it, and its implications for the tumor microenvironment may help in the discovery of new therapeutic targets.

## 1. Introduction

Skin cancer is one of the most common type of cancers, and malignant melanoma is its deadliest form [1,2]. Although the introduction of immune checkpoint inhibitors represents a great advancement in the treatment of melanoma and thus has improved patient prognosis, many patients do not respond to therapy—leaving them with limited treatment options [2,3,4,5,6,7]. Melanoma prognoses remain particularly poor in advanced metastatic disease and in mucosal forms [1,2,8]. One proposed strategy to improve therapeutic efficacy is by targeting the delicate redox balance inside cancer cells.

It is well known that healthy cells require low levels of reactive oxygen species (ROS) for signaling [9]. ROS are naturally produced intracellularly from mitochondrial oxidative phosphorylation (OXPHOS) and from environmental factors, including ultra-violet (UV) radiation and pollutants [10,11]. However, elevated and prolonged amounts of ROS have been associated with tumorigenesis and the progression of cancer, namely by activating pro-tumor signaling pathways, increasing proliferation, cell survival, and genetic instability [12,13,14]. As a result of these conferred advantages combined with their own increased metabolic rates, cancer cells live with increased ROS levels, and thus exist in an inherent state of oxidative stress [12]. However, when ROS levels exceed their threshold of tolerance, they damage vital cellular components, including DNA, lipids, and proteins, and can lead to eventual oxidative stress induced apoptosis [12,15].

Melanoma stands out amongst other solid tumors for having especially high oxidative stress levels, which can be explained from both external and internal factors [16,17]. Due to their physical location, melanoma cells are directly exposed to a myriad of environmental oxidative stress inducing factors, like UV radiation [16,18]. In addition, they also contain melanosomes, which raise intracellular ROS levels through the production of melanin [19,20]. The links between increased oxidative stress levels and malignant melanocyte transformation, as well as melanoma initiation, progression, and metastasis, are well established [19,20,21,22]. As melanoma cells already live with elevated amounts of oxidative stress, they are innately more vulnerable to increases in exogenous ROS levels, which can be therapeutically manipulated, such as with the use of cold atmospheric plasma (CAP).

Plasma, also called the fourth state of matter, can be formed by subjecting gas to an electromagnetic field [23]. Plasma is made of excited ionized gases, which release electromagnetic radiation, including infrared, visible, and UV radiation [23]. The discovery of CAP, non-thermal plasma consisting of neutral atoms, ions, and highly energized electrons, within mild physiological temperatures (less than 40 °C at the point of application) enabled new applications of CAP in biological settings [23,24]. Since then, CAP has been found to have diverse biological effects, including but not limited to tissue regeneration, blood circulation, cell detachment, and disinfection [23,24]. These effects have been principally linked to the increased presence of reactive oxygen and nitrogen species (RONS) resulting from the chemical reaction of CAP with biological tissues and cells [23]. In recent years, CAP has garnered much attention for its anticancer properties, such as its selective cytotoxicity and inhibition of proliferation of cancer cells [25]. These effects have been observed in a wide variety of cancer types, including but not limited to brain, skin, lung, and colorectal cancers [25].

Although the influence of CAP on tumor cells has been extensively studied, relatively little is known regarding how CAP influences the different cells that make up the tumor microenvironment, which can greatly influence therapy response and patient prognosis [26,27]. The present study aimed to address how CAP treatment affects the survival and metabolism of tumor cells, T cells, and macrophages, which, amongst others, compose the melanoma microenvironment. To accomplish this, cells were treated directly with argon and microwave-based CAP for varying treatment times, and CAP induced alterations in cell viability, proliferation, phenotype, and metabolic activity were investigated.

It was found that CAP treatment could significantly decrease the viability and proliferation of melanoma and T cells with T cells being the most affected. When these effects were further investigated, it was found that CAP induced G2/M cell cycle arrest and the consequent apoptosis of melanoma cells. Additionally, CAP treatment influenced the polarization of human monocyte derived macrophages into a “wound-healing” and rather tolerogenic M0/M2 phenotype. CAP treated cells also displayed hallmark signs of mitochondrial stress, including disrupted ATP production and reductions in cell fitness. Antioxidant treated melanoma cells and T cells could partially recover their viability and proliferation in spite of CAP treatment—supporting that the cytotoxic and anti-proliferative effects of CAP treatment likely stem from increased ROS levels.

To better understand how CAP influences the naturally more complex melanoma microenvironment, melanoma cells were cultured to form in vitro 3D tumor spheroids and repeatedly treated with CAP. The resulting spheroids were significantly smaller in size and displayed reductions in cell viability and proliferation when compared to the untreated control. Nevertheless, 3D melanoma spheroids were more resistant to CAP than melanoma cells grown as in vitro 2D monolayers—requiring higher doses of CAP to have its desired anticancer effects.

Collectively, we could show that oxidative stress induced by CAP treatment differentially decreased the viability and proliferation as well as increased the mitochondrial stress levels of various cell types, which comprise the melanoma microenvironment in a CAP treatment time dependent manner. Improving our understanding of oxidative stress, mechanisms to manipulate it, and its implications for the distinct cells in the tumor microenvironment may help in the discovery of new and effective clinical targets.

## 2. Materials and Methods

### 2.1. Cell Culture and Cell Lines Employed

The human melanoma cell lines, Ma-Mel-19 and UKRV-Mel-15a, were cultured with RPMI-1640 (Thermo Fisher Scientific, #31870, Waltham, MA USA) supplemented with 10% FCS (Thermo Fisher Scientific, #10500064), 1% GlutaMAX™ (Thermo Fisher Scientific, #35050038), and 0.1% Primocin (InvivoGen, #ant-pm-2, San Diego, CA, USA). The human glioblastoma cell line, T98G, was cultured with Minimum Essential Medium Eagle (Sigma-Aldrich, #M2279, St. Louis, MO, USA) supplemented with 10% FCS, 1% GlutaMAX™, and 0.1% Primocin.

These cell lines were cultured as previously described [28,29]. Cells were passed every 3–4 days using Trypsin-EDTA (Sigma-Aldrich, #T3924) to detach the cells. UKRV-Mel-15a and Ma-Mel-19 cell lines were obtained from Dr. A. Paschen, in Essen, Germany in 2014 [28,29]. T98G was purchased from the ATCC in 2015. The Cellosaurus accession numbers for each cell line are as follows: Ma-Mel-19 (CVCL_A156), UKRV-Mel-15a (CVCL_A713), and T98G (CVCL_0556). Cell lines were authenticated at Eurofins Genomics in March 2021. The resulting STR profiles matched with the online databases of the German collection of microorganisms and cell cultures (DSMZ) (Available online: https://www.dsmz.de/services/human-and-animal-cell-lines/online-str-analysis, accessed on 30 January 2022) and the Cellosaurus database (Available online: https://web.expasy.org/cellosaurus/, accessed on 30 January 2022). For viability and proliferation experiments, Ma-Mel-19 and UKRV-Mel-15a cells were prelabeled with carboxyfluorescein succinimidyl ester (CFSE) (eBioscience #65-0850-84, San Diego, CA, USA), plated at 100,000 cells per well in 24 well plates (Corning, #3527, Corning, NY, USA), and incubated overnight in a 37 °C 5% CO_2_ incubator. The next day, cells were CAP treated as indicated and incubated another 3 days before analysis.

### 2.2. Isolation and Stimulation of Human T Cell Populations

Buffy coats were obtained from healthy volunteers, with the approval from the local ethics committee (Landesärztekammer Rhineland Palatine No. 837.019.10 (7028), approved on 4 March 2010). PBMC were isolated as described previously [30]. Either CD3^+^, CD4^+^, or CD8^+^ T cells were isolated via CD3, CD4, or CD8 microbeads (Miltenyi Biotec #130-050-101, #130-045-101, #130-045-201, Bergisch Gladbach, Germany) from the company’s respective protocols. For proliferation and viability assays, CD3^+^, CD4^+^ and CD8^+^ T cells were prelabeled with CFSE and plated at 2.5 million cells per well in X-Vivo 15 (Lonza, #BE02-060F, Basel, CH) in 24 well plates, stimulated with 0.5 μg/mL anti-CD3 mAb (clone OKT3, eBioscience) plus 1 μg/mL anti-CD28 mAb (clone 28.2, eBioscience) in the presence or absence of the following antioxidants caffeic acid (Sigma-Aldrich #C0625), rutin-hydrate (rutin) (Sigma-Aldrich #E5143), and resveratrol (Sigma-Aldrich #R5010), as indicated. Plates were incubated overnight in a 37 °C 5% CO_2_ incubator. The next day, cells were CAP treated as indicated and incubated another 3 days before analysis.

### 2.3. Isolation and Polarization of Human Monocyte Derived Macrophages

To generate macrophages, 200 million PBMC were seeded in RPMI1640, containing 1% Glutamax, 0.1% Primocin, and 50 ng/mL M-CSF (ImmunoTools, #11343113, Friesoythe, Germany) in cell culture petri dishes (Corning, #430167) for 1 h in a 37 °C 5% CO_2_ incubator. Afterwards, non-adherent cells were rinsed off by repeated washing of the petri dishes with pre-warmed to 37 °C DPBS (Thermo Fisher Scientific, #14190-094), checked by microscopy. Adherent cells were cultured in the medium described above containing an additional 1% heat inactivated human plasma. Human plasma of ten healthy donors was pooled and centrifuged for 15 min at 800× *g*. The resulting supernatant was centrifuged for 15 min at 1000× *g* to deplete the cellular contents. The pooled plasma was heat inactivated for 20 min at 56 °C. Insoluble denatured protein was removed by centrifugation at 3000× *g* for 15 min at room temperature. The heat inactivated plasma was stored at −20 °C until further use. Cells were harvested by incubating plates with Accutase (Thermo Fisher Scientific, #00-4555-56) for 1 h at 4 °C after 5–7 days of culture. The resulting “M0”-macrophages were polarized with 100 ng/mL LPS (InvivoGen, #tlrl-eblps), 20 ng/mL human IFN-γ (Immunotools, #11343536), and 50 ng/mL M-CSF for an M1-like phenotype and with 20 ng/mL human IL-4 (Immunotools, #11340045) plus 50 ng/mL M-CSF for an M2-like phenotype for 2 days in a 37 °C 5% CO_2_ incubator. For polarization experiments, M0-like macrophages were treated with CAP, as indicated, and were incubated for 2 days before analysis.

### 2.4. Flow Cytometry

Single cell suspensions were stained in 10 μL/sample FACS buffer containing 0.5% human serum albumin (CSL Behring, #10530a/96, King of Prussia, PA, USA), 1 mM EDTA (AppliChem, #A3553, Darmstadt, Germany), 10 μg/mL human IgG (CSL Behring, #EU/1/08/446/001) in DPBS using the following antibodies for flow cytometric analysis. Cells were stained with fixable viability dye FVD506 or FVD780 (Thermo Fisher Scientific, #65-0866-18, #65-0865-14) prior to the surface staining of anti-CD40 (Immunotools, #21270403), anti-CD36 (Miltenyi Biotec, #130-095-480), anti-CD206 (Miltenyi Biotec, #130-100-152), anti-PD-L2 (Miltenyi Biotec, #130-105-829), anti-CD163 (Thermo Fisher Scientific, #A15792), anti-HLA-DR (BioLegend, #307650, San Diego, CA, USA), anti-CD68 (BD Biosciences, #565594, Franklin Lakes, NJ, USA), anti-CD14 (Immunotools, #21279143), anti-CD3 (ImmunoTools, #21620036), anti-CD4 (BD Biosciences, #555347), and anti-CD8 (BioLegend, #344734). For cell cycle analysis, single cell suspensions were washed once with DPBS and stained with Hoechst 33342 (PromoKine, #PK-CA707-40046, Heidelberg, Germany) in DPBS. Apoptotic and necrotic cells were detected by staining single cell suspensions with the FITC Annexin V Apoptosis Detection Kit with PI (BioLegend, #640914) in accordance with the manufacturer’s protocol. Data were acquired on a BD LSRII (BD Biosciences) flow cytometer and analyzed using Cytobank software [31]. Doublets, debris, and dead cell were excluded from analysis. Example gating strategies for melanoma cells, macrophages, and CD3^+^, CD4^+^, and CD8^+^ T cells can be found in Figure S1A–C.

### 2.5. T Cell-Tumor Cell Co-Cultures

Before plating, 24 well plates were precoated with 1 μg/mL anti-CD3 mAb antibody for 1 h in a 37 °C 5% CO_2_ incubator. Ma-Mel-19 cells were either left untreated or irradiated with a dose of 10 Gy from a Gammacell 2000 irradiator (Mølsgaard Medical, Skodsborg, Denmark) to inhibit their proliferation. Ma-Mel-19 cells were then seeded at 120,000 cells per well in anti-CD3 precoated plates and incubated overnight. On the following day, isolated CD4^+^ or CD8^+^ T cells were treated with 1 μg/mL anti-CD28 and seeded with the Ma-Mel-19 cells at 3 million cells per well. On the next day, the co-cultures were treated with 30 s of CAP per well and were incubated another 3 days before analysis.

### 2.6. Seahorse Cell Mito Stress Assays

Seahorse Cell Mito Stress assays were performed according to the manufacturer’s protocol and specifications for a Seahorse XFp Analyzer (Agilent, Santa Clara, CA, USA). The day before the assay, tumor cells (Ma-Mel-19, UKRV-Mel-15a, and T98G) and macrophages were plated at 30,000 cells per well in their standard cell culture medium in Seahorse XFp Cell Culture Miniplates (Agilent, #103025-100). CD4^+^ and CD8^+^ T cells were stimulated with 0.5 μg/mL anti-CD3 mAb and 1 μg/mL anti-CD28 mAb and plated at 500,000 cells per well in their standard cell culture medium in XFp Cell Culture Miniplates. The following day, medium was exchanged for Seahorse assay medium consisting of XF DMEM (Seahorse XF DMEM medium, Agilent, #103575-100) or XF RPMI (Seahorse XF RPMI medium, Agilent, #103576-100) supplemented with 1 mM pyruvate (Agilent, #103578-100), 2 mM glutamine (Agilent, #103579-100), and 10 mM glucose (Agilent, #103577-100). Ma-Mel-19, UKRV-Mel-15a, CD4^+^, and CD8^+^ T cells received XF RPMI medium, whereas T98G received XF DMEM medium. Cells were treated with various amounts of CAP and incubated for 1 h in a 37 °C non-CO_2_ incubator. Meanwhile, Seahorse sensor cartridges (Seahorse XFp FluxPak, Agilent, #103022-100) were loaded with 1.5 μM oligomycin, 1.0 μM FCCP, and 0.5 μM rotenone/antimycin A (Seahorse XFp Cell Mito Stress Test Kit, Agilent, #103010-100). Medium was exchanged once more with the relevant supplemented assay medium for each cell type. Plates were placed into a Seahorse XFp Analyzer and the program “Seahorse XF Mito Stress Test” was ran.

### 2.7. Intracellular ROS Quantification

This assay was performed according to the Reactive Oxygen Species (ROS) Detection Kit protocol (PromoKine, #PK-CA577-K936). Ma-Mel-19 cells were plated at 20,000 cells per well in their standard cell culture medium in flat bottom 96 well cell culture plates (Corning, 96-well Clear Flat Bottom Polystyrene TC-treated Microplates, #3598) and incubated in a 37 °C 5% CO_2_ incubator overnight. The next day, medium was removed, and cells were washed with ROS Assay Buffer, treated with ROS Label (1×), and incubated in a 37 °C 5% CO_2_ incubator for 45 min. ROS Label was removed, and cells were washed once more with ROS Assay Buffer. Cells were treated with various amounts of CAP as indicated and were measured 1 h after treatment. The positive control was treated with ROS Inducer (1×) for 1 h prior to measurement. Fluorescence was recorded at Ex 485 nm/Em 535 nm wavelengths on a Sense Beta Plus microplate reader (Hidex, Turku, Finland) with Plate Reader Software Version 0.5.55.0.

### 2.8. Tumor Spheroid Culture

Ma-Mel-19 cells were prelabeled with CFSE and plated at 2000 cells per well in their standard pre-filtered (0.2 µm) (Pall, Acrodisc sterile filters, #4612, Port Washington, NY, USA) cell culture medium in ultra-low attachment 96 well plates (Thermo Fischer Scientific, Nunclon™ Sphera™ 96-Well, Nunclon Sphera-Treated, U-Shaped-Bottom Microplate, #174925). Plates were centrifuged at 100× *g* for 5 min at 4 °C and then incubated in a 37 °C 5% CO_2_ incubator to promote tumor spheroid formation. After 2 days, spheroids of ~300 µm in diameter had formed and began their different CAP treatment regimens. Medium was exchanged every 3–4 days by carefully removing 100 µL of medium from each well and replacing it with 100 µL of their standard pre-filtered cell culture medium.

### 2.9. Spheroid Digestion

Spheroids were digested into single cell suspensions by first pooling (*n* = 6/condition) spheroids and centrifuging them down at 400× *g* for 5 min at 4 °C to form pellets. Supernatants were discarded, and the pellets were digested in 1 U/mL Dispase II (Sigma-Aldrich, Dispase II, #D4693) in a 37 °C shaker set to 400 rpm for 30–40 min, followed by gentle pipetting to completely dissociate the remaining cells. The reaction was stopped by adding equal parts ice-cold DPBS. The cell suspension was centrifuged down at 400× *g* for 5 min at 4 °C, and the supernatant was discarded. The remaining cell pellets were washed with DPBS and were stained for flow cytometry as indicated.

### 2.10. Spheroid Imaging

Spheroid growth was monitored over time through imaging. Starting at 2 days after plating, spheroids were imaged every 2 days for the duration of each experiment. Images were taken at a 4× magnification using a light inverted microscope (Invitrogen, EVOs FL Color Imaging System, #AMEFC4300, Waltham, MA, USA) at the widest field of view. Images were subsequently analyzed in the analysis program ImageJ to determine the average diameter of each spheroid [32]. The average diameter was calculated by taking the mean of three different diameter measurements per spheroid.

### 2.11. CAP Treatment

Cells were CAP treated with a MiniJet-R from Heuermann HF Technik (Aachen, Germany). The set-up of the device and an explanation of its components can be found in Figure S2A,B. The standard settings used throughout this study were a power level of 4, an argon flow rate of 2 L/min, and a treatment distance of 1 cm from the plasma jet to the surface of the cell culture medium. The duration and frequency of CAP treatment varied depending on the experiment. Cells and spheroids grown in 96 well plates were treated in the center of each well with the plasma jet. However, cells in 24 well plates, due to reasons of increased plate surface area, were CAP treated by manually moving the cell culture plate under the plasma jet to ensure that all cells were equally exposed to CAP.

### 2.12. Statistics

Statistical analysis was performed using GraphPad Prism version 9.0.0 for Windows (GraphPad Software, www.graphpad.com, accessed on 30 January 2022). Results were normalized to the untreated controls as indicated. Bar diagrams display mean ± standard deviation (SD). Statistical significance was determined using ordinary one-way ANOVAs and two-way ANOVAs corrected for multiple comparisons using Dunnett or Tukey tests as well as by paired and unpaired Students *T*-tests, as indicated in the figure legends with * *p* < 0.05, ** *p* ≤ 0.01, *** *p* ≤ 0.001, and **** *p* < 0.0001. Not significant values are indicated by ns and refer to *p* > 0.05.

## 3. Results

### 3.1. CAP Treatment Differentially Affects the Viability and Proliferation of Cells That Make Up the Melanoma Microenvironment

#### 3.1.1. CAP Treatment Reduces the Viability and Proliferation of the Human Melanoma Cell Lines, Ma-Mel-19 and UKRV-Mel-15a, in a Dose-Dependent Manner 

**Figure 1:**
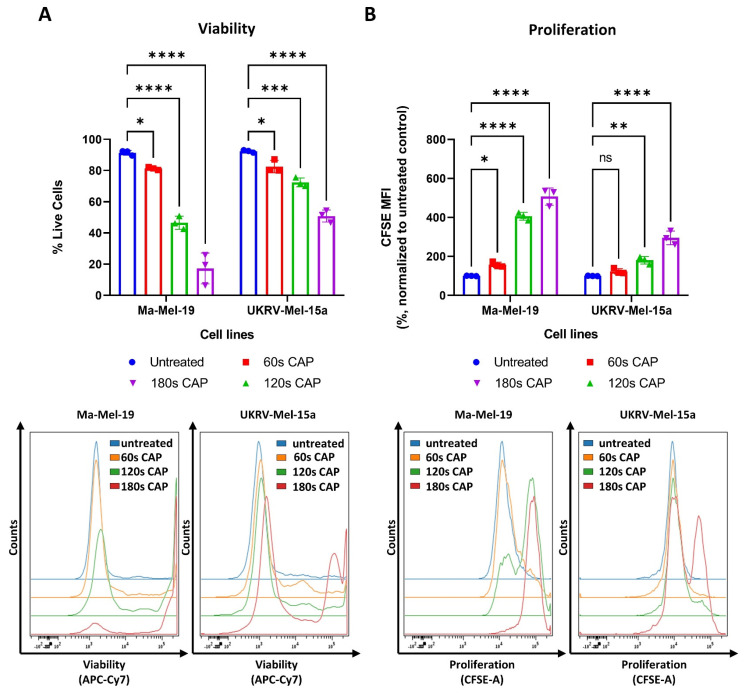

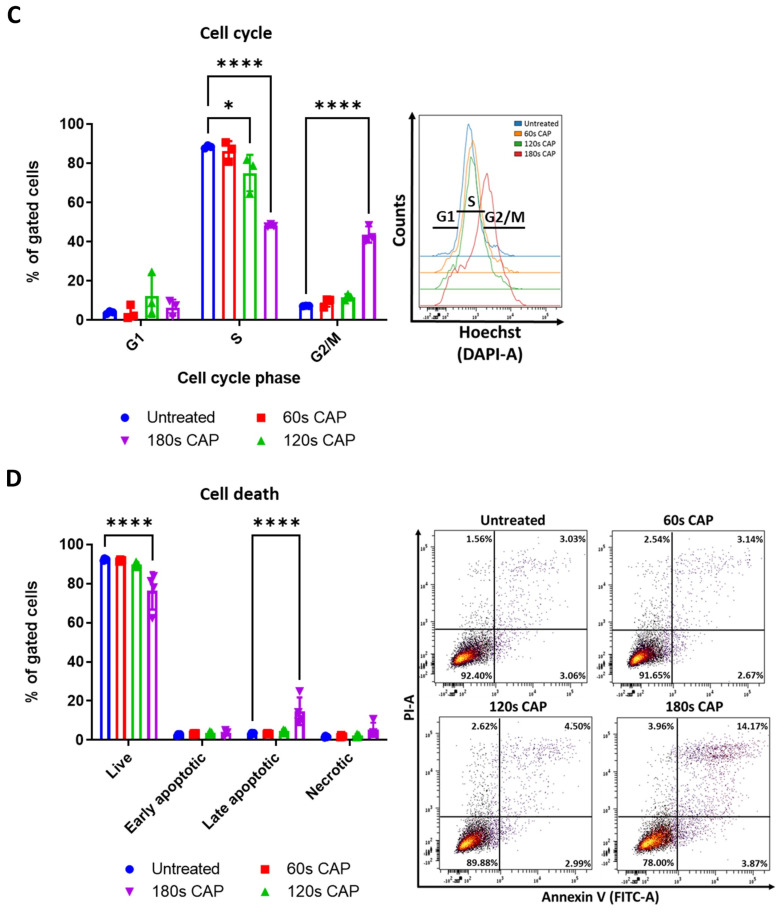
CAP treatment reduces the viability and proliferation of the human melanoma cell lines, Ma-Mel-19 and UKRV-Mel-15a, in a dose-dependent manner. (**A**,**B**) CFSE prelabeled Ma-Mel-19 and UKRV-Mel-15a cells were treated with varying amounts of CAP (60 s, 120 s, 180 s). After 3 days, cells were analyzed via flow cytometry; (**A**) cells were stained for viability. Bar diagram shows the average percentage of live cells measured (*n* = 3) ± SD; (**B**) bar diagram shows the average CFSE mean fluorescent intensity (MFI) measured, normalized to the untreated control (*n* = 3) ± SD; (**C**,**D**) Ma-Mel-19 cells were treated with varying amounts of CAP (60 s, 120 s, 180 s). After 3 days, cells were analyzed via flow cytometry; (**C**) cells were stained with Hoechst. Bar diagram shows the percentage of cells in each gate (*n* = 3) ± SD; (**D**) cells were stained with annexin V and propidium iodide (PI). Bar diagram shows the percentage of cells in each quadrant gate (*n* = 4) ± SD. The contents of each gate are as follows: lower left (LL) live cells, lower right (LR) early apoptotic cells, upper right (UR) late apoptotic cells, and upper left (UL) necrotic cells; histograms and dot plots paired to bar diagrams show one representative result; statistical significance was calculated by performing two-way ANOVAs corrected for multiple comparisons with Dunnett or Tukey tests and is indicated by the asterisks as follows: *, *p* < 0.05; **, *p* < 0.01; ***, *p* < 0.001; ****, *p* < 0.0001.

CAP has been previously shown to have potent inhibitory effects on melanoma cells, including the suppression of cell proliferation and the induction of cell death [33,34]. This study expanded on these results by using the human melanoma cell lines, Ma-Mel-19 and UKRV-Mel-15a, and by evaluating their response to direct CAP treatment by the MiniJet-R, an argon and microwave-based plasma jet. As seen in Figure 1A, significant dose-dependent reductions in cell viability were observed beginning at 60 s of CAP treatment in both cell lines. 

At the highest duration of CAP treatment, 180 s, only 17% of Ma-Mel-19 cells survived, while nearly 50% of UKRV-Mel-15a cells remained alive—demonstrating that melanoma cell lines can display differing sensitivities to CAP treatment. In addition to viability, the effect of CAP on melanoma cell proliferation was also simultaneously examined using CFSE prelabeled cells. As cells divide, the amount of CFSE in the cells proportionally decreases allowing cell proliferation to be analyzed. Therefore, a high CFSE mean fluorescent intensity (MFI) value indicates that cells have proliferated less often than a lower CFSE MFI value. As seen in Figure 1B, a dose-dependent inhibition of cell proliferation was also observed in both cell lines. A significant inhibition of cell proliferation was observed in Ma-Mel-19 cells beginning at 60 s of CAP treatment, while UKRV-Mel-15a required 120 s of CAP treatment to begin to observe a significant inhibition. This demonstrated that UKRV-Mel-15a cells were also more resistant to the anti-proliferative effects of CAP treatment than Ma-Mel-19 cells.

The observed anti-proliferative and cytotoxic effects of CAP on Ma-Mel-19 and UKRV-Mel-15a cells were further investigated to identify the location of cell cycle arrest and the mechanism of CAP induced cell death. It was found that Ma-Mel-19 and UKRV-Mel-15a cells had significantly higher percentages of cells in the G2/M phase following 180 s of CAP treatment than the untreated controls (Figure 1C and Figure S3A). G2/M cell cycle arrest occurs following DNA damage [35]. Therefore, these findings imply that CAP treatment effectively raised the oxidative stress levels of the melanoma cells leading to DNA damage and subsequent cell cycle arrest. These findings also help explain the earlier observed anti-proliferative effects of CAP treatment on melanoma cells (Figure 1B).

To analyze the cytotoxic effects of CAP treatment in more detail, annexin V propidium iodide stainings were performed to differentiate between apoptotic and necrotic cells. It was discovered that both Ma-Mel-19 and UKRV-Mel-15a cells had significantly higher percentages of late apoptotic cells following 180 s of CAP treatment in comparison to the untreated control; however, their percentages of necrotic cells were not significantly altered (Figure 1D and Figure S3B). This showed that the main form of cell death following CAP treatment of Ma-Mel-19 and UKRV-Mel-15a cells is apoptosis. This is well fitting with the G2/M cell cycle arrest results as it is well known that when excessive DNA damage occurs, cells undergo apoptosis [35]. Additionally, these findings of CAP induced G2/M cell cycle arrest and apoptosis are well supported by the literature [25,34].

Altogether, these results show that CAP decreases the viability and suppresses the proliferation of Ma-Mel-19 and UKRV-Mel-15a cells in a dose-dependent manner. Ma-Mel-19 cells were also found to be more sensitive to CAP treatment than UKRV-Mel-15a cells. Furthermore, it was determined that CAP treatment induces G2/M cell cycle arrest and consequent apoptosis of Ma-Mel-19 and UKRV-Mel-15a cells.

#### 3.1.2. CD4^+^ and CD8^+^ T Cells Exhibit a Higher Sensitivity to CAP Treatment Than Melanoma Cells

CAP has been widely hailed as a promising anticancer treatment strategy due to its selective targeting of cancer cells [25]. However, relatively little is known about how CAP affects the other cells that make up the melanoma microenvironment, which can greatly influence the therapy outcome [26,27].

**Figure 2:**
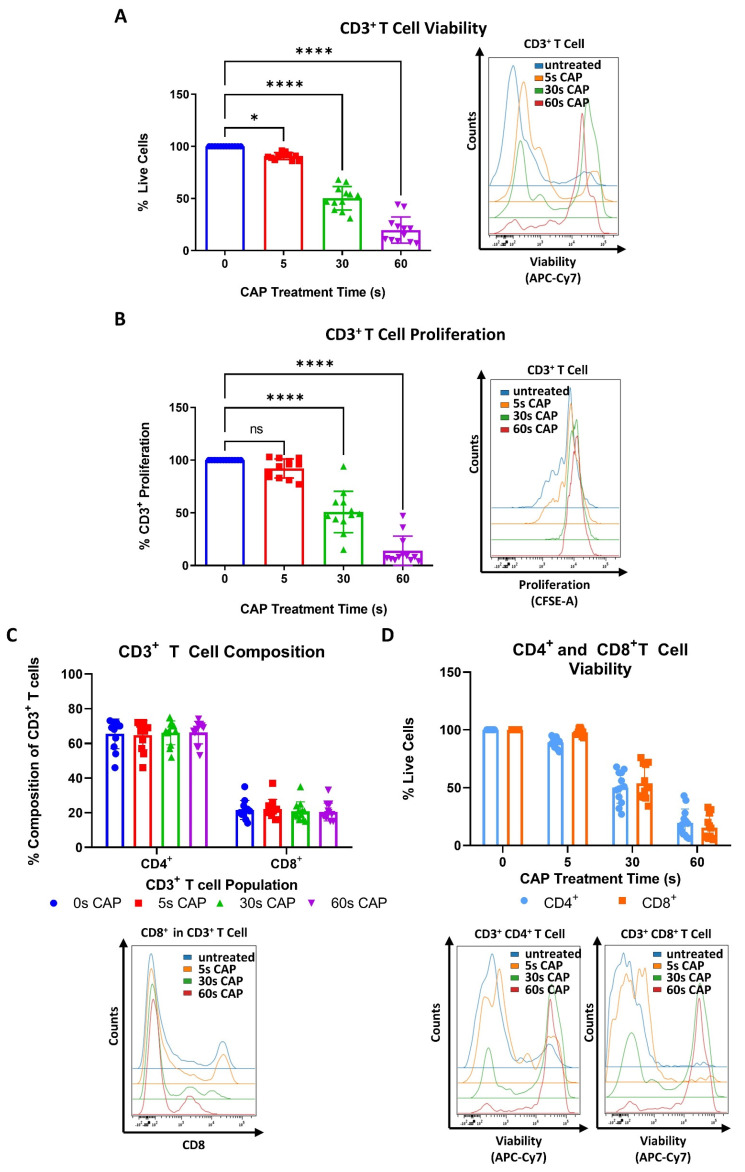

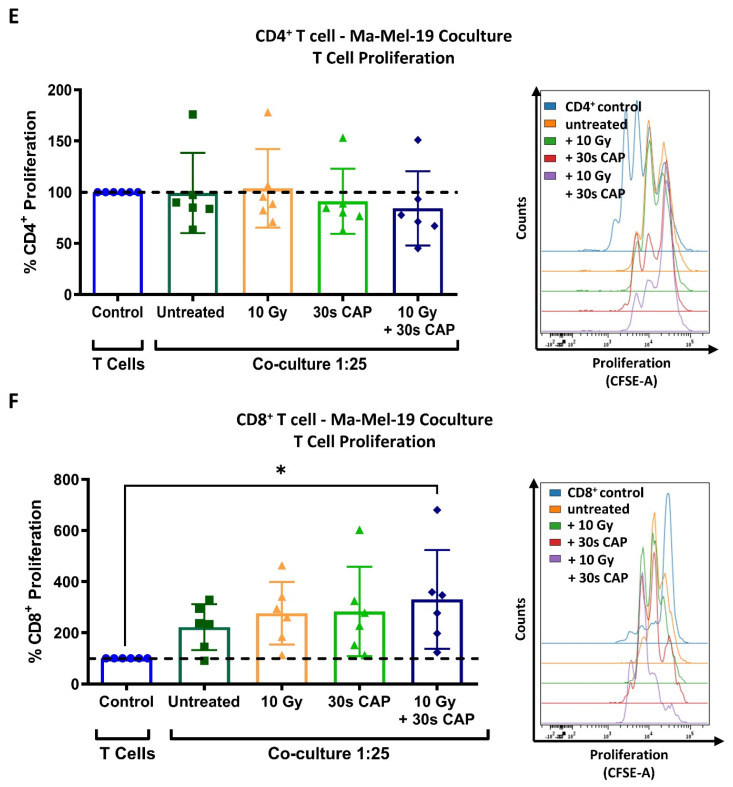
CD4^+^ and CD8^+^ T cells exhibit a higher sensitivity to CAP treatment than melanoma cells. (**A**,**B**) CFSE prelabeled stimulated CD3^+^ T cells were treated with varying amounts of CAP (0 s, 5 s, 30 s, and 60 s). After 3 days, cells were analyzed via flow cytometry; (**A**) cells were stained for viability. Bar diagram shows the average percentage of live cells measured ± SD, normalized to the untreated control of the same donor. Data consist of the pooled results of 4 independent experiments (*n* = 12 donors); (**B**) bar diagram shows the average percentage of proliferating CD3^+^ T cells, ± SD normalized to the untreated control of the same donor. Data consist of the pooled results of 4 independent experiments (*n* = 12 donors); statistical significance was calculated by performing ordinary one-way ANOVA tests corrected for multiple comparisons with Dunnett tests; (**C**,**D**) stimulated CD3^+^ T cells treated with varying amounts of CAP (0 s, 5 s, 30 s, 60 s). After 3 days, cells were analyzed in flow cytometry; (**C**) bar diagram shows the average percentage of CD3^+^CD4^+^ and CD3^+^CD8^+^ T cells in CD3^+^ T cells ± SD, normalized to the untreated control of the same donor. Data consist of the pooled results of 4 independent experiments (*n* = 12 donors); (**D**) CD3^+^CD4^+^ and CD3^+^CD8^+^ T cells were stained for viability. Bar diagram shows the average percentage of live cells measured ± SD, normalized to the untreated control of the same donor. Data consist of the pooled results of 4 independent experiments (*n* = 12 donors); (**E**,**F**) CFSE prelabeled stimulated CD4^+^ and CD8^+^ T cells were grown in co-culture with Ma-Mel-19 cells. Before plating, Ma-Mel-19 cells were either left untreated or were irradiated with a dose of 10 Gy. Co-cultures were treated with 30 s CAP and after 3 days, T cells were analyzed via flow cytometry; (**E**) bar diagram shows the average percentage of proliferating CD4^+^ T cells ± SD, normalized to the T cell as only control of the same donor (*n* = 6 donors). The data consist of the pooled results of 3 independent experiments; (**F**) bar diagram shows the average percentage of proliferating CD8^+^ T cells ± SD, normalized to the T cell as only control of the same donor (*n* = 6 donors). The data consist of the pooled results of 3 independent experiments; histograms paired to bar diagrams show one representative result. Statistical significance was calculated by performing two-way ANOVAs corrected for multiple comparisons with Dunnett tests and is indicated by the asterisks as follows: * *p* < 0.05; **** *p* < 0.0001.

To begin to understand how CAP may affect T cells, which play a pivotal role in the anticancer immune response, stimulated human PBMC isolated CD3^+^ T cells were treated with varying amounts of CAP and assessed for potential changes in viability and proliferative capacity. It was discovered that even with a treatment time of only 5 s CAP, a significant reduction in viability of the whole CD3^+^ T cell compartment could be seen (Figure 2A). 

Viability and proliferation were found to decrease in a dose-dependent manner as CAP treatment times increased (Figure 2A,B). It was also investigated if CAP could influence the composition of CD4^+^ and CD8^+^ T cell sub-populations. However, no significant differences were found (Figure 2C). Additionally, CD4^+^ and CD8^+^ T cells were treated with varying amounts of CAP to see if these T cell sub-populations differentially respond to the cytotoxic effects of CAP. Herein, no significant differences in viability between CD4^+^ and CD8^+^ T cells were observed at any time point (Figure 2D). It was also observed that CD4^+^ and CD8^+^ T cells had a nearly identical response to CAP treatment as the tested whole CD3^+^ T cell compartment (Figure 2A,D).

To further examine how CAP affects T cells in the melanoma microenvironment, co-cultures of CD4^+^ and CD8^+^ T cells cultured with Ma-Mel-19 cells were performed. Interestingly, the proliferation of CD4^+^ T cells grown in co-culture could not be inhibited by 30 s of CAP treatment (Figure 2E) although the proliferation of T cells grown in single cell culture was significantly inhibited following 30 s of CAP treatment (Figure 2B). CD8^+^ T cells also responded differently to 30 s of CAP treatment depending on how they were cultured. When they were co-cultured with Ma-Mel-19 cells, they appeared to exhibit a non-significant increase in proliferation when treated with 30 s of CAP alone. Of note, they showed a significant increase in proliferation when treated with 30 s of CAP and when the Ma-Mel-19 cells were pre-irradiated with 10 Gy to inhibit their proliferation (Figure 2F). Previous studies have supported that CAP can induce immunogenic cell death in cancer cells [36,37,38]. This observed increase in the proliferation of CD8^+^ T cells seems to be suggestive of their potential activation by tumor cell antigens. These antigens were likely released from an increased number of dying tumor cells following combined radiation and CAP treatment, but this must be validated in future studies.

Collectively, these results show that CD3^+^, CD4^+^, and CD8^+^ exhibit a higher sensitivity to CAP treatment than melanoma cells as they required lower doses of CAP to see its cytotoxic and anti-proliferative effects. Additionally, these results suggest that CD4^+^ and CD8^+^ T cells may exhibit differential sensitivities to CAP treatment depending on the way they are cultured—highlighting the large influence an experimental model can have on results.

#### 3.1.3. Macrophages Are Highly Resistant to CAP and Can Be Polarized into a “M0/M2-like” Phenotype

This study also examined the effect of CAP on human monocyte-derived macrophages, another important immune cell type found in the melanoma microenvironment [27]. Resting M0 macrophages were treated with varying amounts of CAP to determine if CAP treatment could affect their viability.

**Figure 3:**
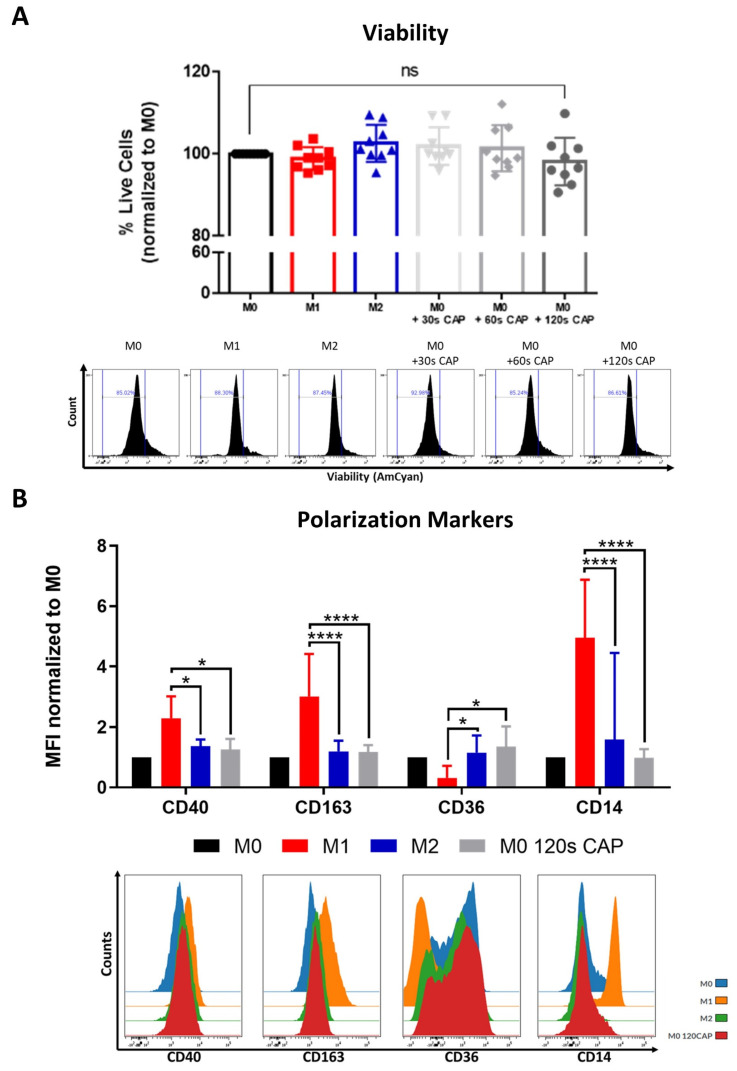
Macrophages are highly resistant to CAP and can be polarized into a “M0/M2-like” phenotype. (**A**,**B**) M0 macrophages were treated with varying amounts of CAP (30 s, 60 s, 120 s). M1 and M2 polarized macrophages served as a control. After 2 days, cells were analyzed via flow cytometry; (**A**) cells were stained for viability. Bar diagram shows the average percentage of live cells measured ± SD, normalized to untreated M0 macrophages, and represents the pooled results of twelve independent experiments (*n* = 12 donors); (**B**) cells were stained with different macrophage polarization markers. Bar diagram shows the average mean fluorescent intensity (MFI) of each measured marker normalized to untreated M0 macrophages and represents the pooled results of twelve independent experiments (*n* = 12 donors); histograms paired to bar diagrams show one representative result. Statistical significance was calculated by performing two-way ANOVAs corrected for multiple comparisons with Tukey tests and is indicated by the asterisks as follows: * *p* < 0.05; **** *p* < 0.0001.

Notably, it was found that even at the highest treatment duration of 120 s CAP, M0 macrophages did not significantly differ in their viability when compared to the untreated M0 control (Figure 3A). 

This indicates that, in this study, they were the cell type tested which was most resistant to the cytotoxic effects of CAP, followed by melanoma cells (Figure 1A) and then by T cells (Figure 2A,D), which were most affected.

Macrophage polarization state, or phenotype, can influence tumor progression, but relatively little is known about the influence of CAP on macrophage polarization. This study aimed to clarify this question by CAP treating M0 macrophages and examining their following polarization state with an extensive macrophage polarization marker panel. M0 macrophages appeared to adopt a rather “M0/M2-like” phenotype following 120 s CAP treatment (Figure 3B). This was supported by the significantly higher expression of the M2 marker, CD36, and significantly lower expression of the M1 markers, CD40, CD163, and CD14. No significant differences in the markers CD206, PD-L2, CD86, and MHCII were observed (Figure S4).

In summary, these results indicate that M0 macrophages are highly resistant to CAP treatment and can be polarized into a more “M0/M2-like” phenotype following CAP treatment.

### 3.2. Cells of the Melanoma Environment Display Varying Signs of Mitochondrial Stress Following CAP Treatment

#### 3.2.1. CAP Induces Mild Metabolic Stress in Cancer Cells

Mitochondrial OXPHOS is an important metabolic process, which is sensitive to the cellular redox balance [10]. Therefore, this study aimed to explore how CAP might influence the mitochondrial functioning of different cells that make up the melanoma microenvironment. This was accomplished by performing Seahorse Cell Mito Stress Tests. This assay provides information on aspects of mitochondrial respiration by measuring the oxygen consumption rate (OCR) and extracellular acidification rate (ECAR) as various electron transport chain inhibitors (oligomycin, rotenone A, antimycin) and the uncoupling reagent, FCCP, are added to the sample. Example OCR and ECAR graphs can be found in Figure S5A,B. Some of the parameters that this assay measures are ATP-production coupled respiration, coupling efficiency, proton leak, and spare respiratory capacity. ATP-production coupled respiration represents the amount of ATP produced by the mitochondria. Coupling efficiency refers to how well the linked reactions of electron carrier oxidation and ADP phosphorylation generate ATP. A proton leak, when protons cross from the inner mitochondrial membrane into the matrix without traveling through the ATP synthase, lowers coupling efficiency and can indicate mitochondrial damage [39,40]. Spare respiratory capacity represents the ability of a cell to respond to increased energetic needs and can be used as an indicator of cell fitness [40].

**Figure 4:**
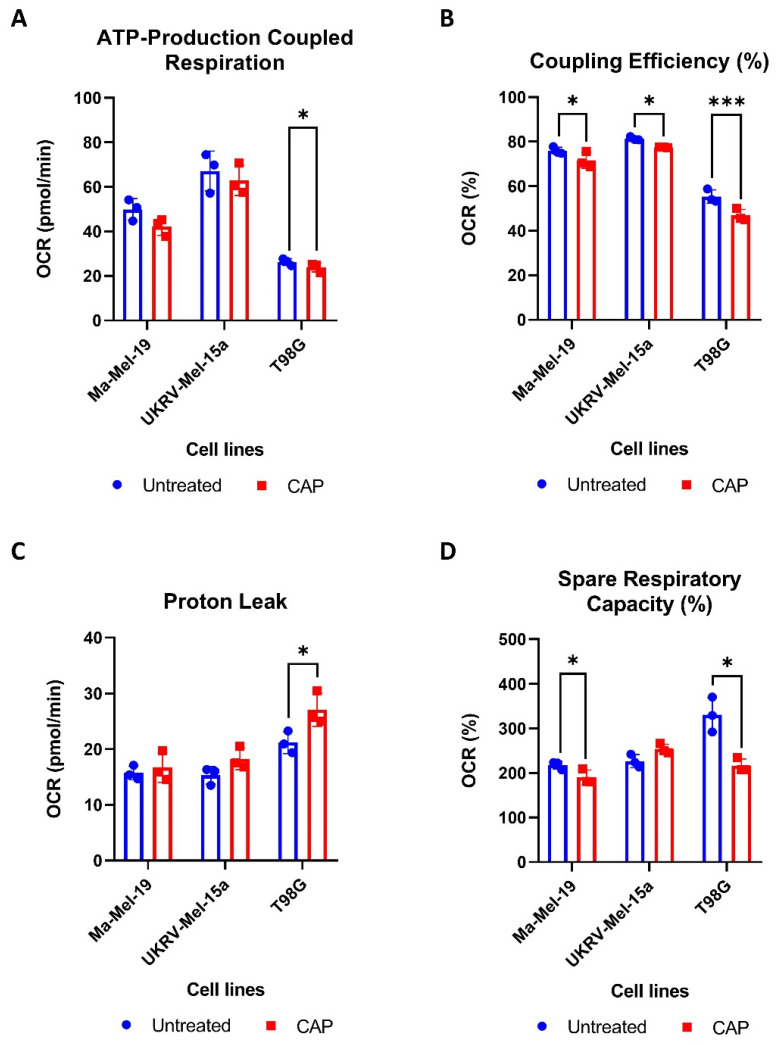
CAP induces mild metabolic stress in cancer cells. (**A**–**D**) Cancer cells were differentially treated with CAP (Ma-Mel-19—120 s CAP, UKRV-Mel-15a—60 s CAP, T98G—60 s CAP) 1 h before undergoing Seahorse Cell Mito Stress Tests. The data from each of the following parameters, (**A**) ATP-production coupled respiration, (**B**) coupling efficiency (%), (**C**) proton leak, and (**D**) spare respiratory capacity (%), show the mean ± SD (*n* = 3); statistical significance was calculated by performing paired Student’s *t*-tests and is indicated by the asterisks as follows: * *p* < 0.05; *** *p* < 0.001.

To determine how CAP acutely affects the mitochondrial functioning of cancer cells, two human melanoma cell lines, Ma-Mel-19 and UKRV-Mel-15a, and a human glioblastoma cell line, T98G, were CAP treated and underwent Seahorse Cell Mito Stress Tests. T98G was tested to see if different cancer types differ in their metabolic response to CAP treatment. UKRV-Mel-15a and T98G were CAP treated for 60s, whereas Ma-Mel-19 cells required 120 s of CAP treatment to begin to observe signs of mitochondrial stress. It was found that ATP-production coupled respiration was significantly reduced in T98G cells, while the melanoma cell lines had only slight but non-significant reductions (Figure 4A). 

All cell lines displayed significant reductions in coupling efficiency following CAP treatment, likely due to an increased proton leak (Figure 4B). It is believed that proton leakage across the inner mitochondrial membrane into the matrix is one of the main causes of incomplete coupling [39]. The proton leak was significantly increased in CAP treated T98G cells, and it appeared to be slightly, but not significantly, elevated in both melanoma cell lines (Figure 4C). Significant reductions in spare respiratory capacity were also observed in Ma-Mel-19 and T98G, likely resulting from disrupted ATP production (Figure 4D). Notably, different cancer cell lines and cancer types tested in this study also displayed varying sensitivities to CAP—highlighting the need to test other cancer entities against CAP in future studies.

Altogether, these results show that CAP induces mitochondrial stress in both melanoma and glioblastoma cells through the disruption of ATP production as evinced from the observed decreases in ATP-production coupled respiration and coupling efficiency as well as the increased proton leak following CAP treatment. It was also determined that cancer cell lines and types exhibit differing sensitives to CAP induced mitochondrial stress.

#### 3.2.2. CD4^+^ and CD8^+^ T Cells Are More Sensitive to CAP Induced Mitochondrial Stress Than Macrophages

The acute influence of CAP on the mitochondrial respiration of CD4^+^ and CD8^+^ T cells and differentially polarized macrophages was also examined. As CD4^+^ and CD8^+^ T cells were found to be highly sensitive to CAP treatment, in terms of viability (Figure 2D), a CAP treatment time of 30 s was selected to be used for the Seahorse Cell Mito Stress experiments. Macrophages were also treated with 30 s of CAP to directly compare how the two different immune cell types respond to CAP treatment.

**Figure 5:**
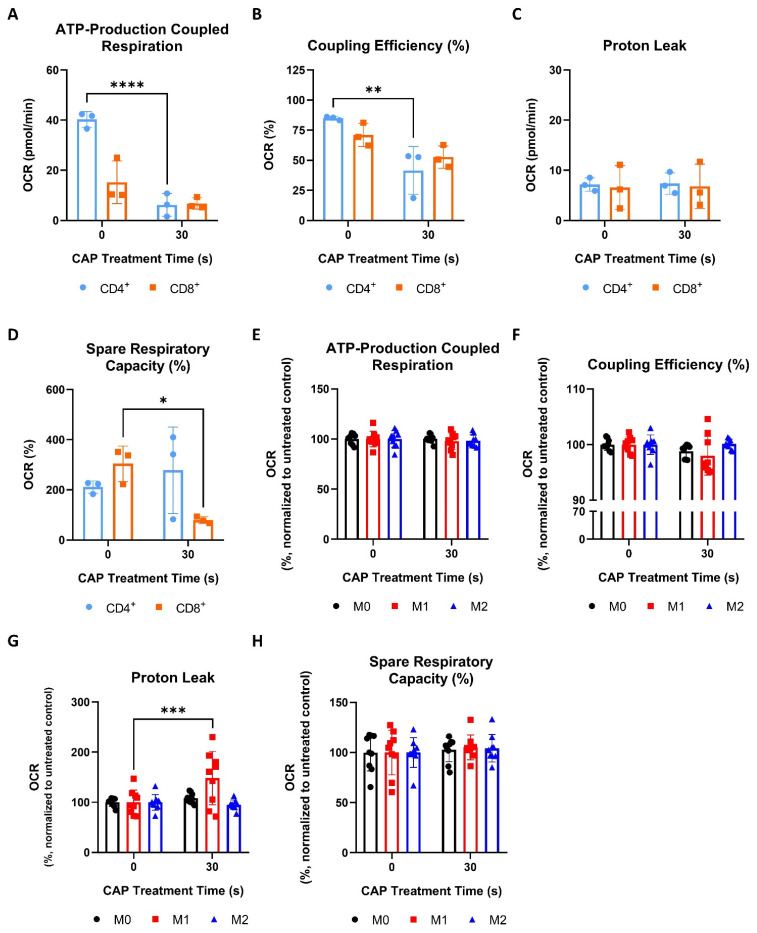
CD4^+^ and CD8^+^ T cells are more sensitive to CAP-induced mitochondrial stress than macrophages. (**A**–**D**) CD4^+^ and CD8^+^ T cells were treated with 30 s of CAP 1 h before undergoing Seahorse Cell Mito Stress Tests. The data from each of the following parameters, (**A**) ATP-production coupled respiration, (**B**) coupling efficiency (%), (**C**) proton leak, and (**D**) spare respiratory capacity (%), show the mean ± SD (*n* = 3 donors); statistical significance was calculated by performing unpaired Student’s *t*-tests; (**E**–**H**) macrophages were treated with 30 s of CAP 1 h before undergoing Seahorse Cell Mito Stress Tests. The data from each of the following parameters, (**E**) ATP-production coupled respiration, (**F**) coupling efficiency (%), (**G**) proton leak, and (**H**) spare respiratory capacity (%), show the mean normalized to the untreated control ± SD (*n* = 9, 3 donors with 3 replicates each); statistical significance was calculated by performing two-way ANOVAs corrected for multiple comparisons with Dunnett tests and is indicated by the asterisks as follows: * *p* < 0.05; ** *p* < 0.01; *** *p* < 0.001; **** *p* < 0.0001.

Like cancer cells (Figure 4A,B), CAP treated CD4^+^ T cells also displayed significant reductions in ATP-production coupled respiration and coupling efficiency (Figure 5A,B). 

However, both CD4^+^ and CD8^+^ T cells did not show any significant increases in proton leaking (Figure 5C). Interestingly, only CD8^+^ T cells had a significant decrease in spare respiratory capacity following CAP treatment (Figure 5D). Collectively, these results show that, although CD4^+^ and CD8^+^ T cells display signs of mitochondrial stress following CAP treatment, they appear to respond to these effects differently. For example, CD4^+^ T cells exhibit a greater disruption of ATP production than CD8^+^ T cells, but only the spare respiratory capacity, or fitness, of CD8^+^ T cells was reduced following CAP treatment (Figure 5A,B,D).

In contrast to T cells (Figure 5A–D), M0, M1, and M2 macrophages were found to be highly resistant to CAP induced mitochondrial stress—exhibiting no significant reductions in ATP-production coupled respiration, coupling efficiency, or spare respiratory capacity following CAP treatment (Figure 5E,F,H). Surprisingly, it was discovered that only M1 macrophages exhibited a significantly increased proton leak and a slight but non-significant reduction in coupling efficiency following CAP treatment (Figure 5F,G).

In summary, these results show that CD4^+^ and CD8^+^ T cells displayed greater signs of acute mitochondrial stress following CAP treatment than macrophages and that sub-populations of T cells and macrophages metabolically respond to CAP treatment differently.

### 3.3. Antioxidant Treatment Mitigates the Effects of CAP on Melanoma and CD3^+^ T Cells

One of the objectives of this study was to see if the cellular response to CAP treatment and thus to CAP induced metabolic alterations could be manipulated. This was especially interesting in the case of T cells to determine if the earlier observed adverse effects of CAP treatment might be reversible. One of the ways CAP is believed to exert its cytotoxic and anti-proliferative effects is through the production of ROS [25,41]. To begin, it was first verified that CAP treatment, from the device used in this study, could increase intracellular ROS levels. This was confirmed by treating Ma-Mel-19 cells with varying amounts of CAP and quantifying the resulting intracellular ROS levels. As seen in Figure S2C, Ma-Mel-19 cells exhibited a dose-dependent increase in intracellular ROS levels following CAP treatment.

Previous studies in cervical cancer cells have shown that the cellular effects of CAP can be combated with various antioxidants [41,42]. To expand upon these results, this study treated cells found in the melanoma microenvironment with a range of easily accessible and naturally occurring antioxidants, belonging to the polyphenol group, alone or in combination with CAP. In more detail, this study tested antioxidants from three different subclasses of polyphenols: phenolic acid (caffeic acid), flavonoids (rutin), and stilbenes (resveratrol) [43]. This was done to provide a more comprehensive understanding of how polyphenol derived antioxidants might be used to provide protection against the potentially undesired effects of CAP treatment.

As described earlier, CAP partially consists of ROS, which are believed to be the main cause of CAP induced cytotoxicity in vitro [25]. Polyphenol derived antioxidants are potent free radical scavengers, which sequester free radicals, such as those found in and produced by ROS. Therefore, it was speculated that they could be used to counteract ROS produced by CAP, and thus reduce the effects of CAP induced oxidative stress. To the best of our knowledge, none of the antioxidants listed above have been tested for their influence on the cellular effects of CAP in the context of the tumor microenvironment, and this combined CAP-antioxidant treatment approach has not been tested before on cells, which specifically make up the melanoma microenvironment. More specifically, this study tested CD3^+^ T cells and melanoma cells (Ma-Mel-19 and UKRV-Mel-15a) with this CAP-antioxidant combination therapy approach as they were found to be the most sensitive to CAP treatment in this study.

**Figure 6:**
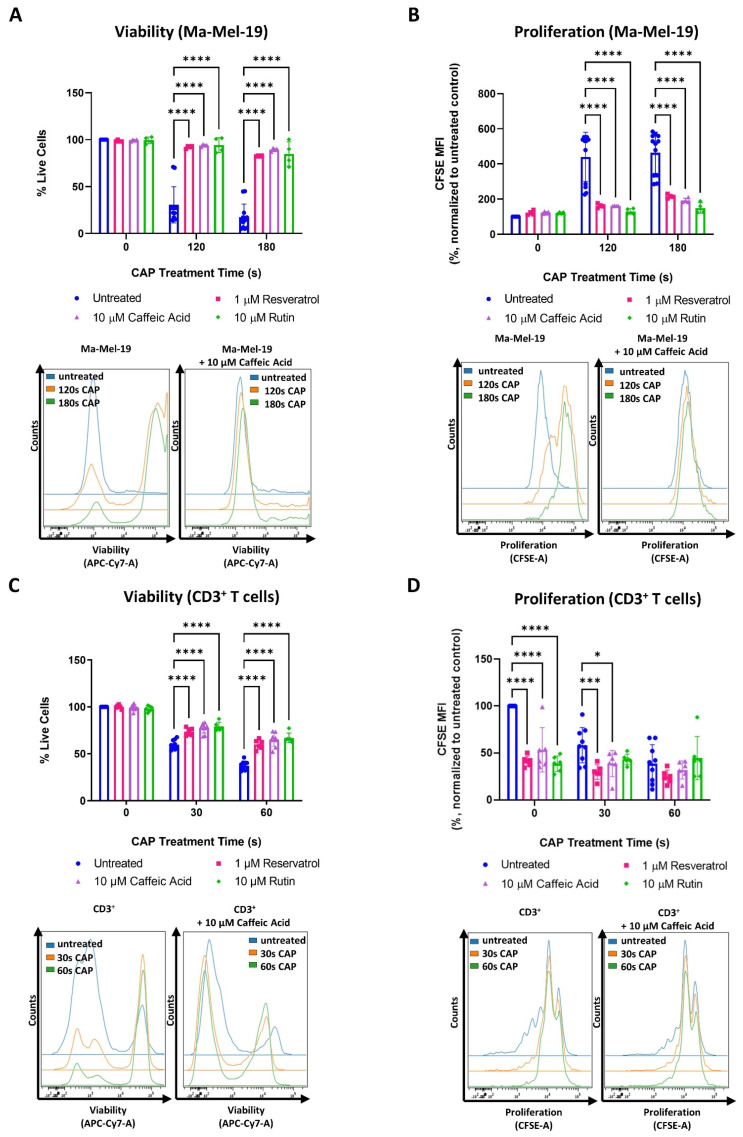
Antioxidant treatment mitigates the effects of CAP on melanoma and CD3^+^ T cells. (**A**,**B**) CFSE prelabeled Ma-Mel-19 cells were plated in the presence of various antioxidants and differentially treated with CAP (0 s, 120 s, 180 s). After 3 days, cells were analyzed via flow cytometry; (**A**) Ma-Mel-19 cells were stained for viability. Bar diagram shows the average percentage of live cells measured ± SD and represents the pooled results of 3 independent experiments (*n* = 12/untreated, *n* = 4/antioxidant); (**B**) bar diagram shows the average CFSE mean fluorescent intensity (MFI) measured, normalized to the untreated control ± SD and represents the pooled results of 3 independent experiments (*n* = 12/untreated, *n* = 4/antioxidant); (**C**,**D**) CFSE prelabeled stimulated CD3^+^ T cells were plated in the presence of various antioxidants, and differentially treated with CAP (0 s, 30 s, 60 s). After 3 days, cells were analyzed via flow cytometry; (**C**) CD3^+^ T cells were stained for viability. Bar diagram shows the average percentage of live cells measured ± SD and represents the pooled results from 3 independent experiments (total replicate size: *n* = 9/untreated, *n* = 6/antioxidant); (**D**) bar diagram shows the average percentage of proliferating CD3^+^ T cells normalized to the untreated control from the same donor (*n* = 9/untreated, *n* = 6/antioxidant) ± SD and represents the pooled results from 3 independent experiments; histograms paired to bar diagrams show one representative result. Statistical significance was calculated by performing two-way ANOVAs corrected for multiple comparisons with Dunnett tests and is indicated by the asterisks as follows: * *p* < 0.05; *** *p* < 0.001; **** *p* < 0.0001.

It was found that the tested concentrations of resveratrol, caffeic acid, and rutin alone had no effect on the viability and proliferation of Ma-Mel-19 cells (Figure 6A,B). 

However, when Ma-Mel-19 cells were treated with a combination of CAP and antioxidants, they were found to be significantly more viable and proliferative than when treated with CAP alone. In fact, CAP-antioxidant combination treated cells nearly recovered their viability and proliferation to untreated levels.

Similar effects were also observed with the cell line, UKRV-Mel-15a. None of the tested antioxidants had a significant effect on cell viability or proliferation when compared to the untreated control (Figure S6A,B). However, with the application of antioxidants, the viability and proliferation of CAP treated UKRV-Mel-15a cells could nearly be restored to untreated levels. When the different antioxidants tested were compared to each other, no significant differences in their effects on Ma-Mel-19 and UKRV-Mel-15a cells, in terms of viability and proliferation, were observed at any time point (Figure 6A,B and Figure S6A,B).

The same tested concentrations of resveratrol, caffeic acid, and rutin were also found to have no effect on the viability of CD3^+^ T cells (Figure 6C), but CAP-antioxidant combination treatment could only partially restore the viability of CD3^+^ T cells. In contrast to the Ma-Mel-19 and UKRV-Mel-15a cells, every antioxidant tested was found to inhibit the proliferation CD3^+^ T cells (Figure 6D). When CD3^+^ T cells received a combination of CAP-antioxidant therapy, their proliferation was not only unable to be recovered but significantly decreased, as in the cases of 30 s CAP-resveratrol and 30 s CAP-caffeic acid combination therapy.

Collectively, these results support that CAP partially mediates its cytotoxic and anti-proliferative effects through its generation of ROS as viability (melanoma and CD3^+^ T cells) and proliferation (melanoma) of CAP treated cells could be recovered following treatment with various polyphenol derived antioxidants.

### 3.4. Ma-Mel-19 Cells Are More Resistant to Repeated CAP Treatment When Grown as 3D Tumor Spheroids

**Figure 7:**
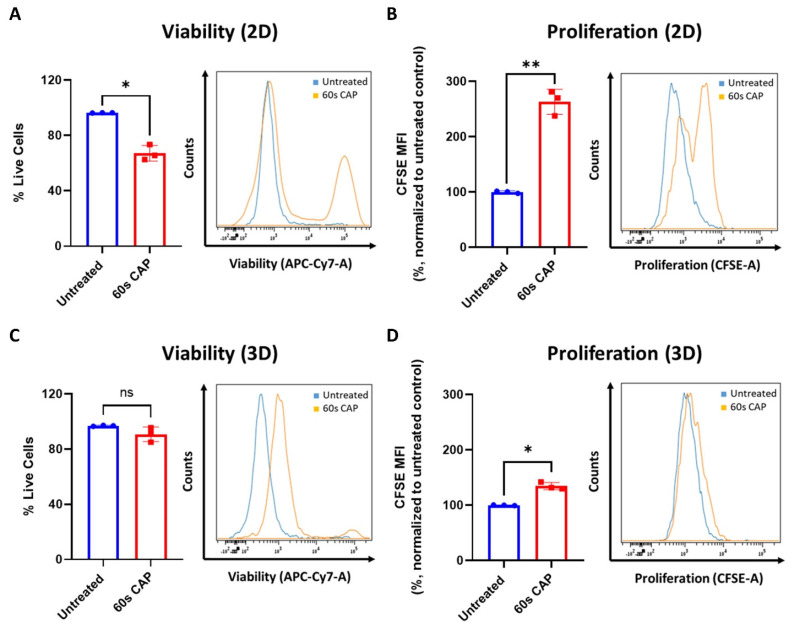

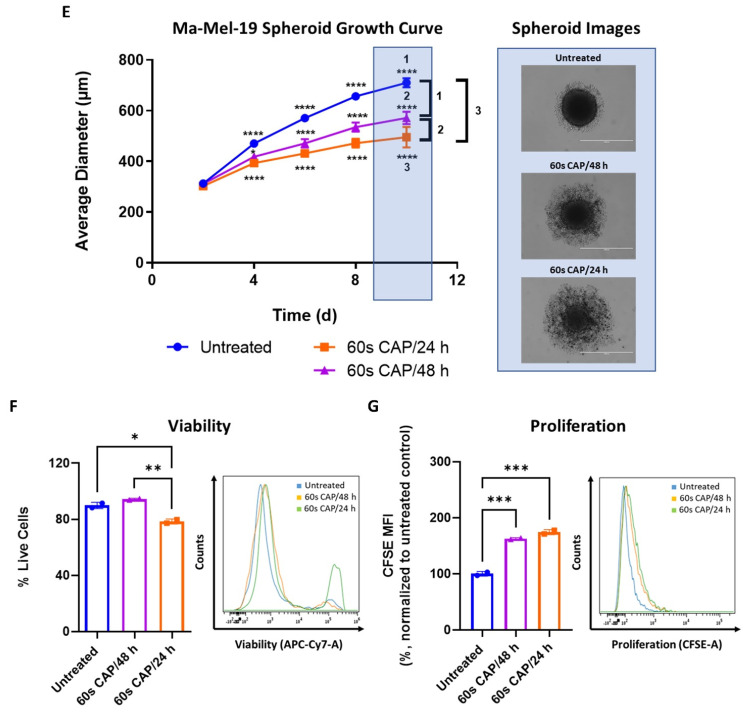
Ma-Mel-19 cells are more resistant to repeated CAP treatment when grown as 3D tumor spheroids. (**A**,**B**) CFSE prelabeled Ma-Mel-19 cells were grown in 2D in vitro monolayers and treated with 60 s CAP for 3 consecutive days. On the day after, cells were analyzed in flow cytometry; (**A**) cells were stained for viability. Bar diagram shows the average percentage of live cells measured (*n* = 3) ± SD; (**B**) bar diagram shows the average CFSE mean fluorescent intensity (MFI) normalized to the untreated control (*n* = 3) ± SD; statistical significance was calculated by performing paired Student’s *t*-tests; (**C**,**D**) CFSE prelabeled Ma-Mel-19 cells were grown as 3D in vitro tumor spheroids and were treated with 60 s CAP for 3 consecutive days. On the day after, spheroids were pooled (*n* = 6 spheroids/replicate of each condition, *n* = 3), digested, and analyzed in flow cytometry; (**C**) spheroids were stained for viability. Bar diagram shows the average percentage of live cells measured ± SD; (**D**) bar diagram shows the average CFSE MFI measured, normalized to the untreated control ± SD; statistical significance was calculated by performing paired Student’s *t*-tests; (**E**–**G**) CFSE prelabeled Ma-Mel-19 cells were grown as 3D in vitro tumor spheroids. Spheroids were treated with 60 s of CAP every day (60 s CAP/24 h) or every other day (60 s CAP/48 h) for 8 consecutive days. On the day after, spheroids were pooled (*n* = 6 spheroids/replicate of each condition, *n* = 2), digested, and analyzed in flow cytometry; (**E**) spheroids were imaged (*n* = 9) every other day to monitor their growth. Images were analyzed to determine spheroid diameter. Data shown represents the average spheroid diameter of each condition overtime ± SDs. Representative images of spheroids with or without CAP treatment at day 10 are shown; (**F**) spheroids were stained for viability. Bar diagram shows the average percentage of live cells measured ± SD; (**G**) bar diagram shows the average CFSE MFI measured, normalized to the untreated control ± SD; histograms paired to bar diagrams show one representative result. Statistical significance was calculated by performing ordinary one-way ANOVA tests corrected for multiple comparisons with Tukey tests and is indicated by the asterisks as follows: *, *p* < 0.05; **, *p* < 0.01; ***, *p* < 0.001; ****, *p* < 0.0001.

As shown in Figure 2B,E,F, T cells responded differently to CAP depending on whether they were grown in single cell culture or were co-cultured with Ma-Mel-19 cells. To investigate differential responses to CAP treatment depending on the experimental model utilized further, Ma-Mel-19 cells were cultured in both 2D in vitro monolayers and as 3D in vitro tumor spheroids to better model the more complex melanoma microenvironment. Normally, in vitro studies almost exclusively use cells cultured as homogenous adherent 2D monolayers or as free-floating suspension cells [44]. Although these models are rightfully widely used, cells are grown in highly artificial conditions, such as having minimal contact to each other and the surrounding extracellular matrix. Three-dimensional tumor spheroids offer a more physiologically relevant in vitro test system as spheroids share many of the characteristics of in vivo tumors, such as gradients in oxygen and nutrient availability and a resulting hypoxic and necrotic core [44,45]. These test systems were directly compared to each other by treating Ma-Mel-19 cells grown in 2D monolayers and as 3D tumor spheroids with an identical CAP treatment regime, consisting of 60 s of CAP every 24 h for 3 consecutive days. This repeated CAP treatment plan was implemented to enhance the effects of CAP treatment on Ma-Mel-19 cells. It was found that Ma-Mel-19 cells grown in 2D were significantly more sensitive to repeated CAP treatment, as shown by their significant reductions in viability and proliferation (Figure 7A,B). 

However, 3D Ma-Mel-19 spheroids did not display a significant reduction in viability following the same repeated CAP treatment, but a significant reduction of cell proliferation was observed, albeit to a lesser extent, as seen in 2D (Figure 7C,D). In summary, this demonstrates that Ma-Mel-19 cells grown as 3D tumor spheroids are more resistant to CAP therapy than when grown as 2D monolayers.

Due to this observed increased resistance of Ma-Mel-19 spheroids to CAP, the duration of CAP therapy was extended to 8 days to see if the spheroids could be made more susceptible to CAP treatment. Two different CAP treatment approaches were used, where spheroids were treated every day (60 s CAP/24 h) or every other day (60 s CAP/48 h) with CAP, to explore how the frequency of CAP application could affect the spheroids. Spheroids began to receive CAP treatment starting at 2 days after plating, once they were completely formed and had an average diameter of ~300 μm. It is important to note that all conditions had no significant differences in their average diameters on day 2 before the start of CAP treatment (Figure 7E). At day 4, spheroids treated with either CAP treatment regime already exhibited significantly smaller average diameters than the untreated control. It was also seen that spheroids treated with CAP daily were significantly smaller than spheroids treated with CAP every other day. Differences in average diameter between untreated versus CAP treated spheroids only became larger with time, as did the differences between spheroids treated with CAP daily versus every other day (Figure 7E). Although CAP treated spheroids grew slower than the untreated control, CAP treatment was not successful in halting the growth of the spheroids completely. It is interesting to note that CAP treated spheroids also exhibited noticeable alterations in their morphology, namely an accumulation of surrounding loosely attached cells and the cores of the spheroids were visibly less dense and compact (Figure 7E). These features became more pronounced with more frequent applications of CAP (60 s CAP/24 h). This suggests that prolonged CAP treatment is seemingly able to damage the structure of Ma-Mel-19 spheroids.

On day 10, spheroids were digested and analyzed for alterations in their viability and proliferation. Only spheroids treated every day with CAP, displayed significant reductions in viability (Figure 7F). However, proliferation in both CAP treatment regimens was significantly inhibited in comparison to the untreated control (Figure 7G).It is important to note that although increasing the duration and frequency of CAP treatment resulted in less viable and proliferative cells than spheroids treated only 3 times with CAP (Figure 7C,D), the effects of extended CAP treatment on 3D spheroids (Figure 7F,G) were markedly less effective, in terms of reducing viability and proliferation, than those observed in 2D culture (Figure 7A,B).

Taken together, these results demonstrate that Ma-Mel-19 cells are more resistant to CAP when grown as 3D tumor spheroids. However, by increasing the duration and frequency of CAP treatment, it was possible to reduce the size of Ma-Mel-19 spheroids and the viability and proliferation of the cells that make it up.

## 4. Discussion

The discovery of cold atmospheric plasma (CAP), or plasma within physiological temperatures (<40 °C), enabled new applications of CAP in biological settings. When CAP is applied to biological tissues, the main reactive components it generates are RONS and UV radiation [23]. These reactive components lead to complex biochemical interactions with cells and result in a diverse range of biological effects, including blood coagulation, sterilization, cell detachment, and wound healing [24]. In recent years, CAP has attracted much attention as an innovative therapeutic option for cancer treatment. CAP has been shown to have multiple anticancer effects, such as selectively inhibiting proliferation, increasing DNA damage, and subsequently inducing apoptosis of cancer cells [13,25,46]. This selective effect of CAP on tumor cells is not yet completely understood, but one theory is that they are more sensitive to CAP induced oxidative stress than other cells in the body [25].

Normally cells require low levels of ROS for signaling, and ROS are naturally generated as by-products of OXPHOS [10]. However, prolonged elevated ROS levels have been linked to cancer promoting effects, including activating pro-tumor signaling pathways, increasing proliferation, cell survival, and genetic instability [12,13]. Due to these advantages and their own increased rates of metabolism, cancer cells constantly live with elevated ROS levels and thus exist in an inherent state of oxidative stress. Cancer cells cope with these elevated levels by increasing their production of antioxidant proteins [12]. However, when ROS levels rise above their tolerance threshold, ROS can damage cellular components, including lipids, DNA, and proteins, and lead to oxidative stress induced apoptosis [12,15]. This makes tumors cells innately more vulnerable to increases in exogenous ROS levels, which can be therapeutically manipulated, such as through the use of CAP.

CAP can be applied directly administered to cells of the tumor microenvironment or be indirectly administered as plasma treated liquids [47]. A drawback of direct CAP application is that it remains physically limited to superficial tissues; however, it is possible to use it as a post-operative treatment for the surgical field to eliminate remaining tumor cells [24]. Indirect treatment through the application of plasma pretreated liquids allows the potential systemic application to larger tumors and metastases. However, concerns exist regarding the safety of this method of application [24]. Nevertheless, CAP has shown promising results in a wide range of cancer types in in vitro studies, in several in vivo studies, and in a few clinical studies [24,25,36,48].

The present study addressed how CAP treatment can affect the survival and metabolism of cells that compose the melanoma microenvironment, including tumor cells, T cells, and macrophages. It was found that with increasing CAP treatment times, viability and proliferation of melanoma and T cells decreased in a dose-dependent manner. When these CAP induced cytotoxic and anti-proliferative effects were further investigated, it was found that CAP treatment led to G2/M cell cycle arrest and apoptosis of melanoma cells (Figure 1C,D and Figure S3A,B). This is highly suggestive that CAP treatment led to extensive DNA damage, resulting from increased oxidative stress levels, and subsequent apoptosis of melanoma cells. Although this study did not confirm the presence of DNA damage, it is well established that CAP can increase DNA damage, inhibit cell proliferation, and induce apoptosis of cancer cells, making these collective findings in agreement with previous works [13,25,46].

The findings of this study also showed that T cells were more sensitive to CAP than tumor cells. Previous studies have shown that T cells require low levels of ROS for activation, proliferation, and differentiation; however, with too high levels, they inhibit proliferation, damage DNA, and induce apoptosis [49]. The results of this study are in agreement with these tell-tale signs of too high ROS exposure. The CAP treatment times used in this study seem to have consistently produced too high ROS levels to be tolerated by CD3^+^, CD4^+^, and CD8^+^ T cells, as seen by the decreases in viability and proliferation following 30 s of CAP exposure (Figure 2A,B,D). This indicates that the minimum CAP treatment time (60 s) required to have noticeable effects on the tested melanoma cell lines (Figure 1A,B) already would result in the severe cytotoxic and anti-proliferative effects on CD3^+^, CD4^+^, and CD8^+^ T cells grown in a single cell culture (Figure 2A,B,D). CD4^+^ and CD8^+^ T cells also exhibited various signs of mitochondrial stress following 30 s of CAP treatment, whereas the different melanoma cell lines tested required double (UKRV-Mel-15a) or even quadruple (Ma-Mel-19) the dose of CAP to begin to show signs of mitochondrial stress. These results collectively challenge the established field of thought that CAP selectively targets tumor cells as CD3^+^, CD4^+^, and CD8^+^ T cells were consistently more sensitive to CAP treatment in this study.

This difference in observed selectivity likely results from the chosen cell comparison. In the past, many studies have compared the sensitivity of cancer cells to CAP with their corresponding healthy cell type [25,33,46]. Although these comparisons provide helpful information, it is important to also consider how CAP influences the immune cell compartment in the tumor microenvironment, which can greatly influence therapy response and patient prognosis [26,27]. The discovery that CD3^+^, CD4^+^, and CD8^+^ T cells were more sensitive to CAP treatment than tumor cells in this study is disconcerting. It is worth noting that differences in resistance to oxidative stress have been observed amongst different T cell subpopulations. For instance, regulatory T cells are believed to be more resistant to oxidative stress due to their elevated production of antioxidants and their capability to persist in high numbers in the tumor microenvironment [50,51]. Future studies should take care to examine how CAP influences the different T cell sub-populations, especially Tregs, and their implications for therapy response.

To see if the undesired effects of CAP treatment on CD3^+^ T cells could be counteracted, CD3^+^ T cells and melanoma cells were treated with various polyphenol derived antioxidants to attempt to neutralize CAP induced ROS. As a brief background, polyphenols are generally easily oxidized by free radicals, especially when they contain ortho or para diphenol functional groups. Delocalized electrons in their aromatic phenol rings then allow for the formation of more stable phenol radicals, which help end the radical transfer chain [43]. This leads to the sequestration of highly reactive free radicals, which can readily react with and damage vital cellular components. Due to the well-known free radical reducing function of polyphenol derived antioxidants, it was tested in this study if they could be used to neutralize ROS produced by CAP. Although this approach could partially recover the viability of CD3^+^ T cells, all the tested antioxidants inhibited their proliferation with and without CAP treatment (Figure 6C,D). It was also found that both of the melanoma cell lines tested responded significantly better to antioxidant treatment than CD3^+^ T cells—nearly recovering their viability and proliferation to untreated levels (Figure 6A,B and Figure S6A,B). These collective results indicate that this antioxidant intervention strategy to CAP treatment was only partially successfully. Overall, these results agreed with other studies as they showed that one of the ways CAP exerts its cytotoxic and anti-proliferative effects is through its generation of ROS as these effects could be directly negated with the application of various antioxidants [25,41]. Future studies should test alternative antioxidants to see if the anti-proliferative effects on CD3^+^ T cells observed in this study can be avoided. 

This study also provided valuable information on the influence of CAP on the viability and polarization on macrophages. Out of all the cell types tested in this study, M0 macrophages were found to be the most resistant to the cytotoxic effects of CAP, displaying no significant decreases in viability even after 120 s of CAP treatment (Figure 3A). These results agree with a previous study, which showed that the survival of monocyte derived macrophages was unaffected following 30 s of CAP treatment [52]. However, macrophages still exhibited potentially detrimental effects from CAP treatment, in terms of their polarization state and mitochondrial stress levels. Our study demonstrated that M0 macrophages could be effectively polarized into a more “M0/M2-like” phenotype following CAP treatment. Previous studies have examined the effects of CAP on macrophage polarization with conflicting results, likely stemming from their different macrophage sources and analyzed polarization markers [52,53]. Our study provided much needed clarity by staining with a more expansive macrophage polarization panel and by utilizing more physiologically relevant human monocyte derived macrophages in place of THP-1 differentiated macrophages [53]. Tumor associated macrophages, which aid in cancer progression, are generally described to exhibit more of a M2-like phenotype, making the implications of CAP treatment on the tumor microenvironment concerning [26]. Nevertheless, CAP induced M2 polarization is in accordance with the well-known wound healing and skin regeneration effects of CAP described in the literature [23,24].

This study reported for the first time that M1 macrophages exhibited a greater sensitivity to CAP induced oxidative stress as indicated by their increased proton leak, suggestive of mitochondrial damage. This finding is in alignment with the results of Griess et al., who found that M1 macrophages have higher amounts of intracellular ROS and are more sensitive to increases in ROS levels than M2 macrophages [54]. Usually, M1 macrophages are considered to be desirable in cancer treatment as they can help mediate inflammatory anti-tumor immune responses [55]. Therefore, future studies should investigate the long-term effects of CAP treatment on macrophages, especially in more complex experimental models, such as 2D and 3D macrophage-tumor cell co-cultures and in in vivo studies, to determine whether CAP may lead to an unwanted increase in M2 macrophages in the tumor microenvironment.

Although the results of this study showed potentially concerning effects of CAP on T cells and macrophages in vitro, initial studies using murine melanoma models have shown promising effects of CAP in vivo. Depending on the different study designs and treatment modalities, significant inhibition of tumor growth, reduction of tumor burden, increased survival rates, and even tumor eradication have been observed [56,57,58,59]. In addition, it was reported that CAP did not damage the skin at the point of application [56,57]. Further analysis of the in vivo murine melanoma microenvironment has remained limited to introductory histology stainings. Although they have revealed the presence of necrotic tumor tissue following CAP treatment, in-depth studies regarding the effect of CAP on the cells in the in vivo murine melanoma microenvironment have yet to be performed [57,58]. However, the importance of the immune cell compartment in in vivo murine melanoma tumor clearance following CAP treatment has already been supported. In more detail, initial studies have shown an increase in tumor immunogenicity following CAP treatment in in vivo murine melanoma models, including the upregulation of MHC-I and calreticulin [60,61]. Altogether, this suggests that CAP could be used to induce immunogenic cell death of in vivo murine melanoma cells.

The results of this study remain difficult to reconcile when compared to the findings of in vivo murine melanoma models as an in-depth analysis of the immune cell compartments found in the in vivo melanoma microenvironment has yet to be performed. However, it is clear to see that CAP plays a highly complex role in the melanoma microenvironment. On one hand, it can seemingly support tolerogenic immune responses, such as the induction of M2 macrophages, as observed in this study. On the other hand, it is also linked to inducing inflammatory immunogenic cell death of melanoma cells [60,61]. Unraveling how these seemingly conflicting immunological effects play out in more complex in vivo experimental models, including the identification of favorable immune cell populations and their implications for anti-tumor immune responses, should be an essential focal point for future studies.

It was also found that the different cancer cell lines and types tested in this study exhibited varying sensitives to CAP induced oxidative stress in terms of their respective viability, proliferation, and mitochondrial stress levels. Between the two melanoma cell lines tested, Ma-Mel-19 was found to be more sensitive to CAP treatment, in terms of viability and proliferation (Figure 1A,B), while the glioblastoma cell line T98G displayed greater signs of mitochondrial stress than both the tested melanoma cell lines following CAP treatment (Figure 4A–D). Nonetheless, differences between other cancer cell lines and types and their sensitivity to CAP treatment have been reported previously [62,63]. The underlying mechanisms for differential sensitivities of cancer cells to CAP must be investigated in future studies.

Only two other known studies have examined the effects of CAP on melanoma spheroids [64,65]. The findings of this study validated the cytotoxic and the inhibitory effects of CAP on melanoma spheroid size, as observed by Hasse et al. [65]. However, this study differed from Hasse et al. and Sagwal et al. as it examined the effects of repeated long-term CAP treatment on melanoma spheroids [64,65]. Whereas Hasse et al. and Sagwal et al. only treated their spheroids once with CAP and incubated them for up to 1 day before analysis, this study explored how extended and repeated CAP treatment could affect the long-term growth, viability, and proliferation of melanoma spheroids [64,65]. This study also tested different CAP treatment regimens. It was found that increasing the frequency of CAP application (60 s/24 h) and the duration of treatment (8 consecutive days) led to the most significant reductions in spheroid size, viability, and proliferation, providing valuable treatment information to inform future in vivo studies (Figure 7E–G). Additionally, by using a prolonged treatment duration, CAP induced morphological alterations on melanoma spheroids could be uncovered, demonstrating that long-term CAP application can greatly disrupt the structure of melanoma spheroids (Figure 7E). Future work aims to characterize these morphological changes further and to examine the effects of CAP on 3D immune and tumor cell co-cultures.

Previous studies have shown that 3D tumor spheroids can exhibit increased drug resistance when compared to cells grown in 2D monolayers [45]. Herein, the inherent structure of spheroids themselves appears to play an important role. For instance, spheroids naturally develop gradients in oxygen and nutrient availability leading to a size dependent formation of hypoxic and necrotic cores [45,66,67,68]. This hypoxic environment leads to the activation of genes associated with drug resistance and cell survival and a decrease in ROS formation, thus reducing therapeutic efficacy [45,69,70,71,72]. This study was one of the first of its kind to evaluate if melanoma spheroids are more resistant to CAP induced oxidative stress than melanoma cells grown in 2D monolayers. By using an identical CAP treatment regime, it was discovered that cells grown as 3D tumor spheroids were much more resistant to the cytotoxic and anti-proliferative effects of CAP (Figure 7A–D).

The choice of experimental model was also seen to affect the proliferation of CAP treated CD4^+^ and CD8^+^ T cells. When T cells were grown in a single cell culture, their proliferation was greatly inhibited following 30 s of CAP treatment (Figure 2B). However, when they were co-cultured with melanoma cells and treated with the exact same dose of CAP, their proliferation was seemingly unaffected (Figure 2E,F). Taken together, it becomes evident that cells grown in 2D single cell cultures, the least physiologically relevant experimental model tested in this study, were the most sensitive to CAP treatment. This observed decrease in the therapy effectiveness as the experimental models increased in their complexity has been attributed to one of the reasons many promising in vitro drug candidates fail in vivo studies [45,73]. Future studies should evaluate potential drug candidates and therapeutic strategies in more complex in vitro cell culture models, such as tumor spheroids, before proceeding to in vivo studies to limit the unnecessary use of experimental animals and to increase translational efficacy [44,45,74].

The results from this study also suggest that CAP may be suitable for other treatment applications. In more detail, the findings, that CAP could polarize M0 macrophages towards a tolerogenic M0/M2 phenotype and the observed heightened sensitivity of inflammatory M1 macrophages to CAP induced mitochondrial stress, all seem to suggest that CAP may enrich M2 macrophages and in turn support wound healing and anti-inflammatory responses. As mentioned earlier, CAP is already used in clinical dermatology for wound healing and skin regeneration therapies [24,25]. However, alternative applications of CAP for other inflammatory diseases are much less characterized. Due to the inherent physical limits of CAP application, superficial inflammatory skin conditions, such as psoriasis, eczema, and atopic dermatitis, represent particularly promising diseases that may profit from CAP treatment. Evidence of the beneficial effects of CAP for the treatment of psoriasis and atopic dermatitis has already been shown. Initial case studies in psoriasis have shown a beneficial effect of CAP treatment in clearing plaques and preventing recurrence [75,76]. Additionally, an initial pilot study with atopic dermatitis patients found that CAP could reduce the disease severity [77]. Altogether, these results support the promise of CAP treatment for inflammatory conditions, which should be an important focus of future studies.

Collectively, this study provided a comprehensive insight into how CAP induced oxidative stress affects the viability, proliferation, phenotype, and metabolism of different cells that comprise the melanoma microenvironment. Herein, the sensitivity to CAP treatment differed between T cells, macrophages, and tumor cells. In general, T cells and tumors cells were more sensitive to oxidative stress than macrophages. However, we could also show that CAP influenced the polarization of M0 macrophages to a “M0/M2-like” phenotype and that M1 macrophages exhibit a heightened sensitivity to CAP induced mitochondrial stress. Additionally, we could also demonstrate that CAP treatment could effectively inhibit the growth and kill melanoma cells in both 2D and 3D in vitro cell culture models in a dose-dependent manner. Improving our understanding of oxidative stress, ways we can manipulate it, and its implications on the cells that comprise the tumor microenvironment may help in the discovery of new and effective clinical targets.

## 5. Conclusions

In conclusion, we could show that oxidative stress, induced by CAP, could significantly influence viability, proliferation, and mitochondrial respiration of cells, which comprise the melanoma microenvironment. This study contributed to our understanding of oxidative stress, its implications on the survival and metabolism of cells in the tumor microenvironment, and how it can be manipulated in the hopes of contributing to the identification of new and effective therapeutic strategies.

## Data Availability

Not applicable.

## Supplementary Materials

The following are available online at https://www.mdpi.com/article/10.3390/cells11060930/s1, Figure S1: Gating strategies, Figure S2: MiniJet-R and intracellular ROS quantification, Figure S3: UKRV-Mel-15a cell cycle and cell death data, Figure S4: Non-significant macrophage polarization markers, Figure S5: Example Seahorse OCR and ECAR data, Figure S6: UKRV-Mel-15a antioxidant data.
