# Supplementary material for: Oxidative Stress Differentially Influences the Survival and Metabolism of Cells in the Melanoma Microenvironment
Source: Cells. 2022 Mar 8;11(6):930. doi: 10.3390/cells11060930 (PMC8946823; doi:10.3390/cells11060930)
Supplement: Supplementary file 1 [file cells-11-00930-s001.zip › cells-1600573-supplementary.pdf]

Figure S1: Gating strategies

A: Melanoma cells (Ma-Mel-19, UKRV-Mel-15a)

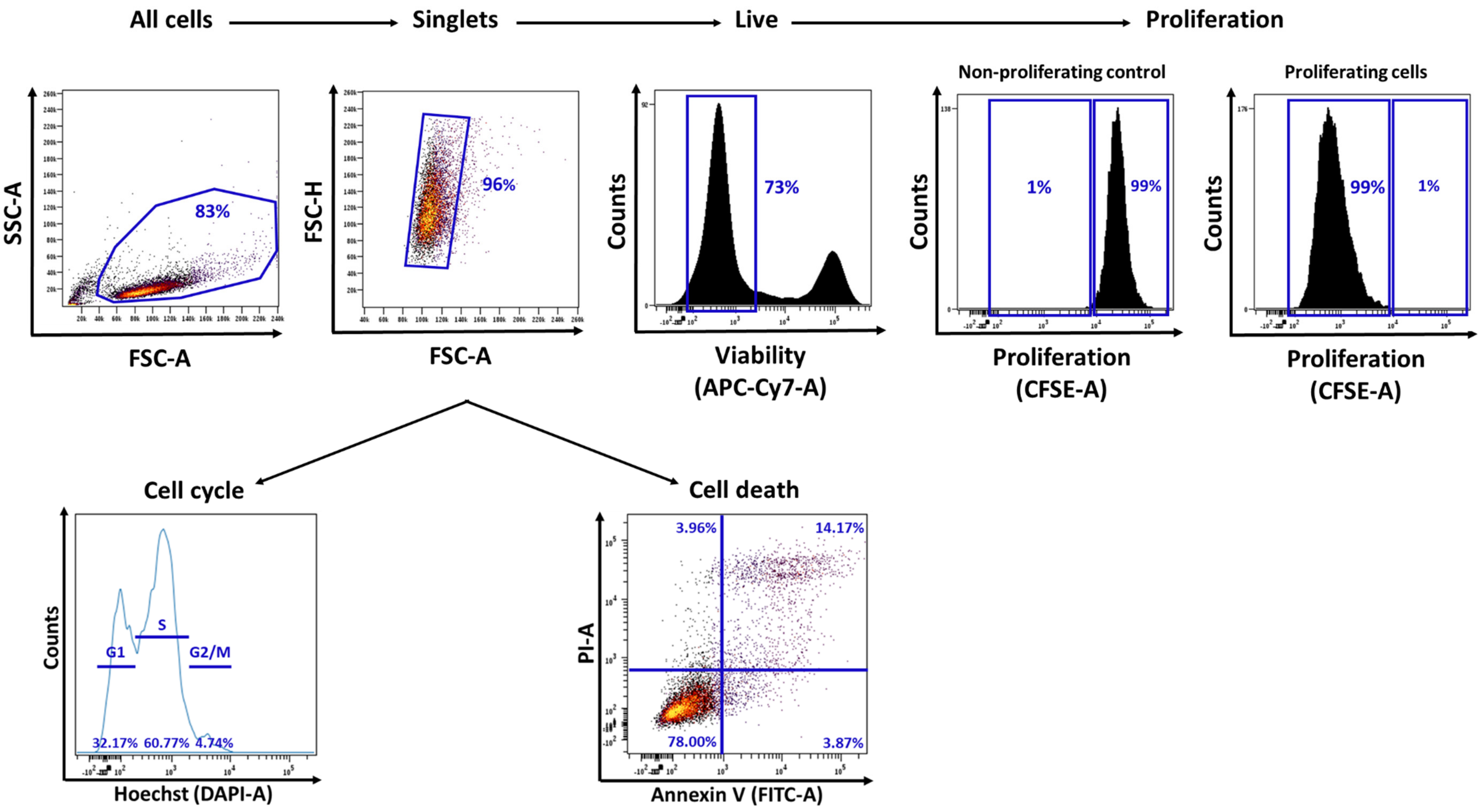

Figure S1: continued

B: Macrophages (M0, M1, M2)

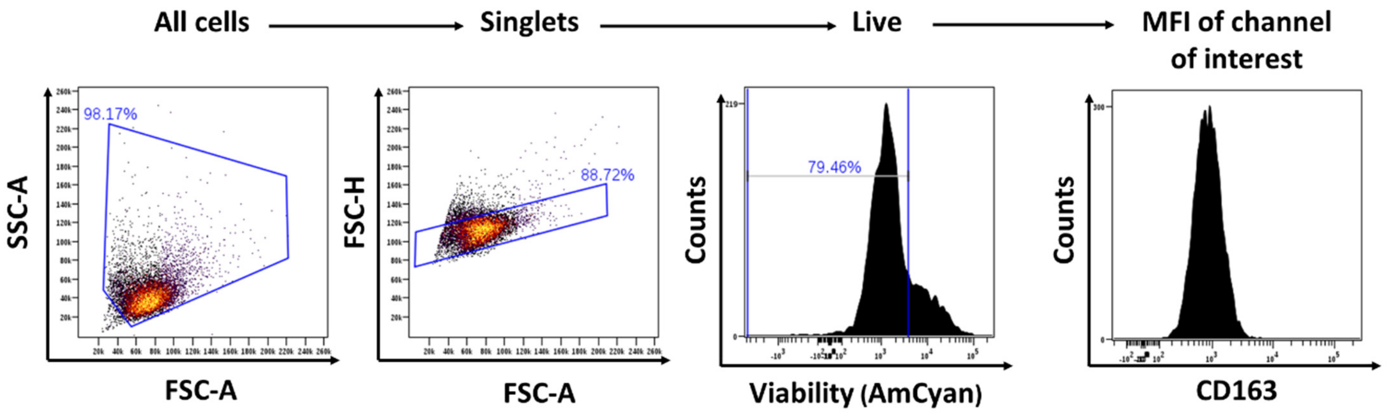

Figure S1: Model gating strategies used for melanoma (**A**), macrophages (**B**), and $CD3^+CD4^+$ and $CD3^+CD8^+$ T cells (**C**) For cell death analysis using annexin V and propidium iodide (**A**), the gating contents of each quadrant are as follows: lower left (LL) live cells, lower right (LR) early apoptotic cells, upper right (UR) late apoptotic cells, and upper left (UL) necrotic cells. In addition, the macrophage gating strategy (**B**), shows one gated polarization marker, CD163, as an example. This same gating strategy was applied for the other measured macrophage polarization markers; however, the channel of interest changed depending on the measured marker; Debris, doublets, and dead cells were excluded from analysis.

Figure S1: continued

C: CD3⁺CD4⁺ and CD3⁺CD8⁺ T cells

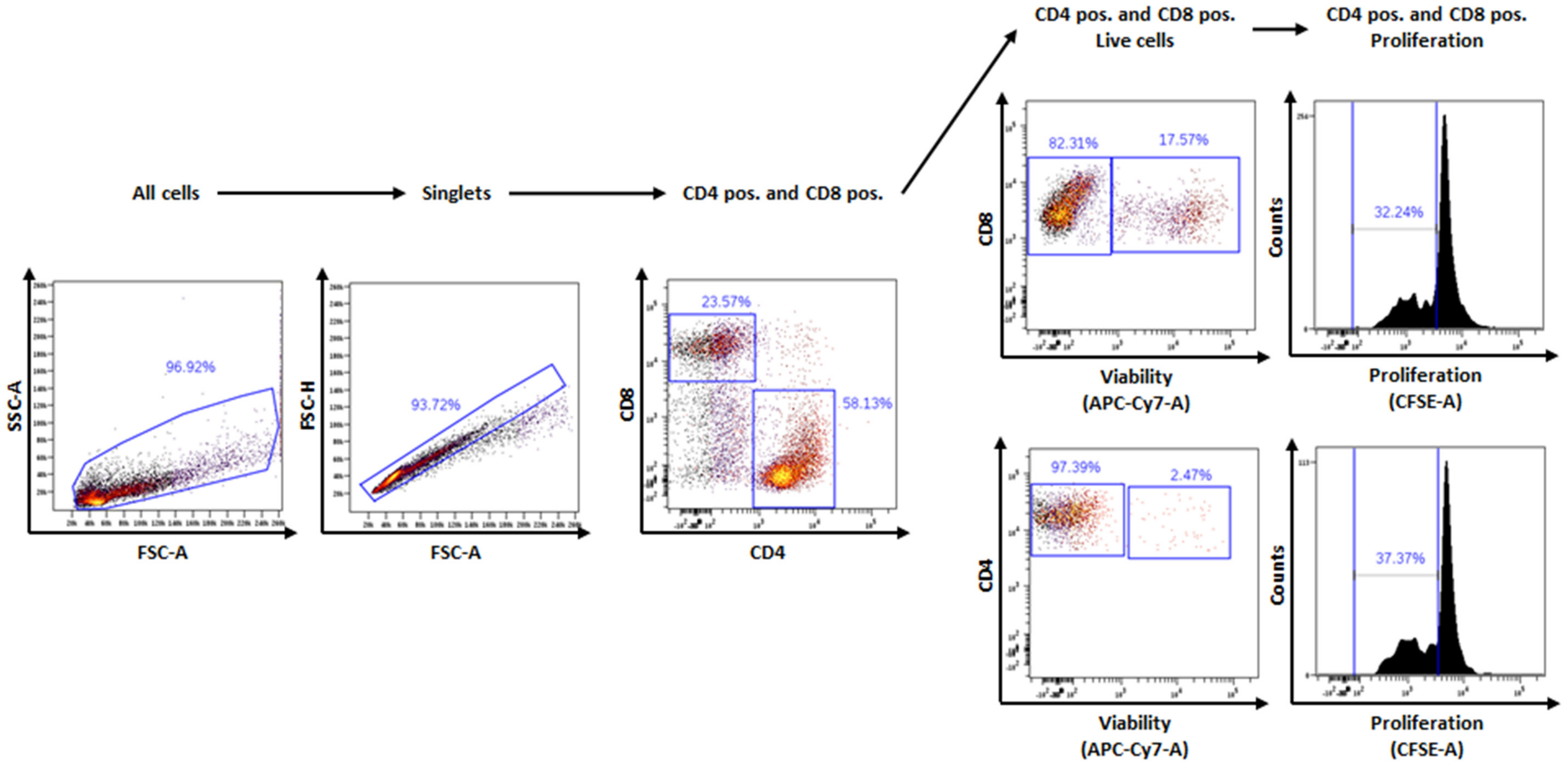

Figure S2: MiniJet-R and intracellular ROS quantification

A: MiniJet-R device

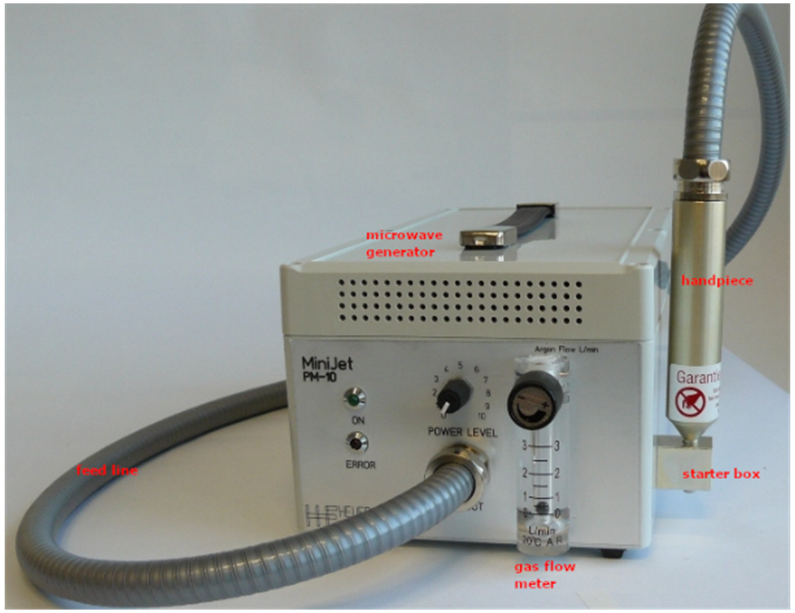

C:

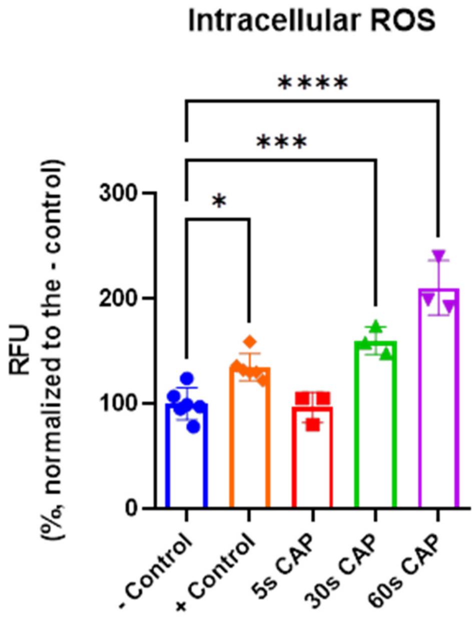

B: MiniJet-R in operation

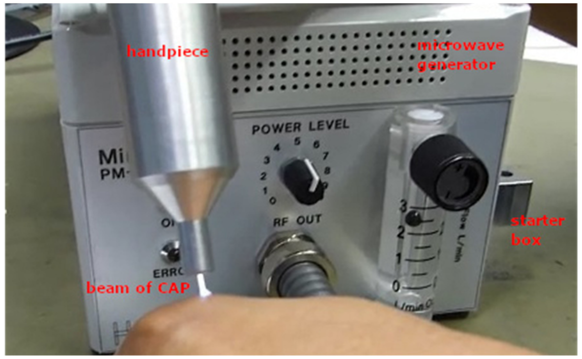

Figure S2. (**A**) The MiniJet-R, from Heuermann HF Technik, used to produce CAP in this study, is comprised of a microwave generator and handpiece. In the feed line, the process gas argon and electromagnetic waves with a frequency of 2.45 GHz are transported to the handpiece. Gas flow can be monitored with the gas flow meter, and the starter box is necessary to ignite the plasma (Picture is taken from [76]); (**B**) Example image of the MiniJet-R in use. A beam of CAP applied to the tissue of a human hand (Picture is taken from [76]); (**C**) Ma-Mel-19 cells were treated with varying amounts of CAP (5s, 30s, 60s) and their intracellular ROS levels were measured 1 h after treatment. The positive control indicates cells treated with a control ROS Inducer, while the negative control represents untreated cells. Bar diagram shows the average intracellular ROS levels in relative fluorescent units (RFU) normalized to the untreated control (n=6/control, n=3/CAP) ± SD; Statistical significance was calculated by performing ordinary one-way ANOVAs corrected for multiple comparisons with Tukey tests and is indicated by the asterisks as follows: *, $P < .05$; ***, $P < .001$; ****, $P < .0001$.

Figure S3: UKRV-Mel-15a cell cycle and cell death data

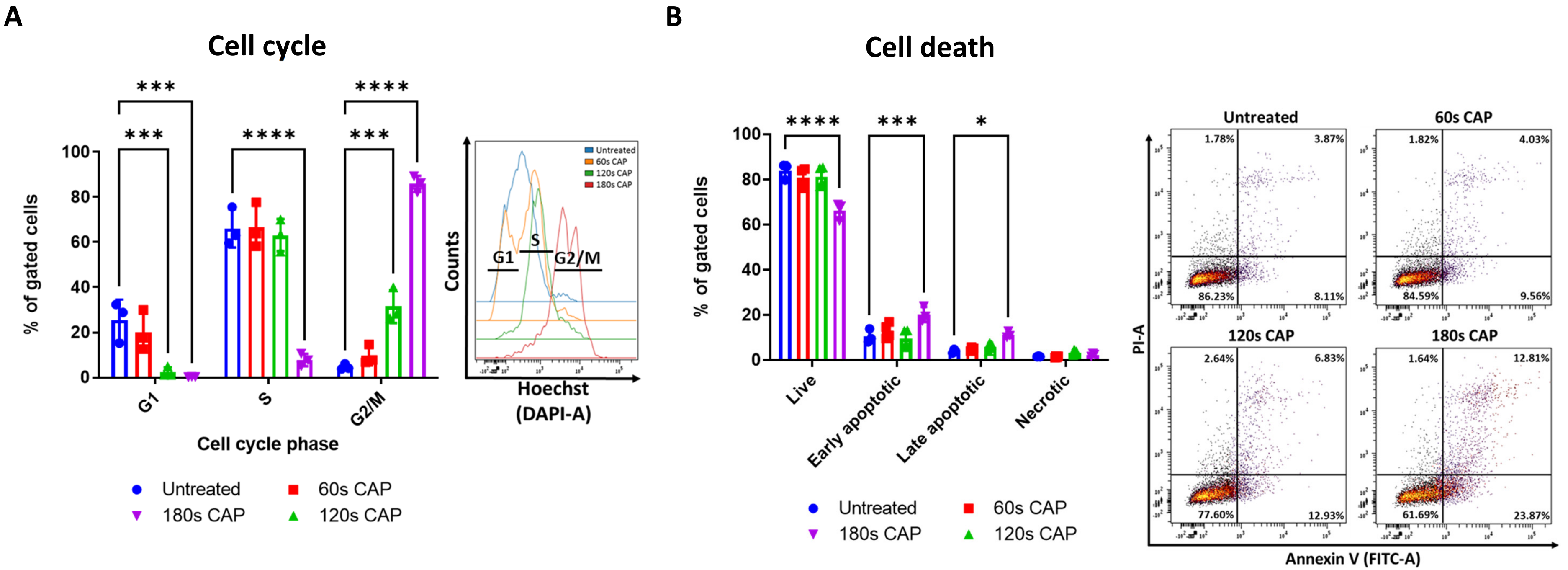

Figure S3. (**A-B**) UKRV-Mel-15a cells were treated with varying amounts of CAP (60s, 120s, 180s). After 3 days, cells were analyzed via flow cytometry; (**A**) Cells were stained with Hoechst. Bar diagram shows the percentage of cells in each gate (n=3) ± SD; (**B**) Cells were stained with annexin V and propidium iodide (PI). Bar diagram shows the percentage of cells in each quadrant gate (n=4) ± SD. The contents of each gate are as follows: lower left (LL) live cells, lower right (LR) early apoptotic cells, upper right (UR) late apoptotic cells, and upper left (UL) necrotic cells; Histograms and dot plots paired to bar diagrams show one representative result; Statistical significance was calculated by performing two-way ANOVAs corrected for multiple comparisons with Tukey tests and is indicated by the asterisks as follows: *, $P < .05$; ***, $P < .001$; ****, $P < .0001$.

Figure S4: Non-significant macrophage polarization markers

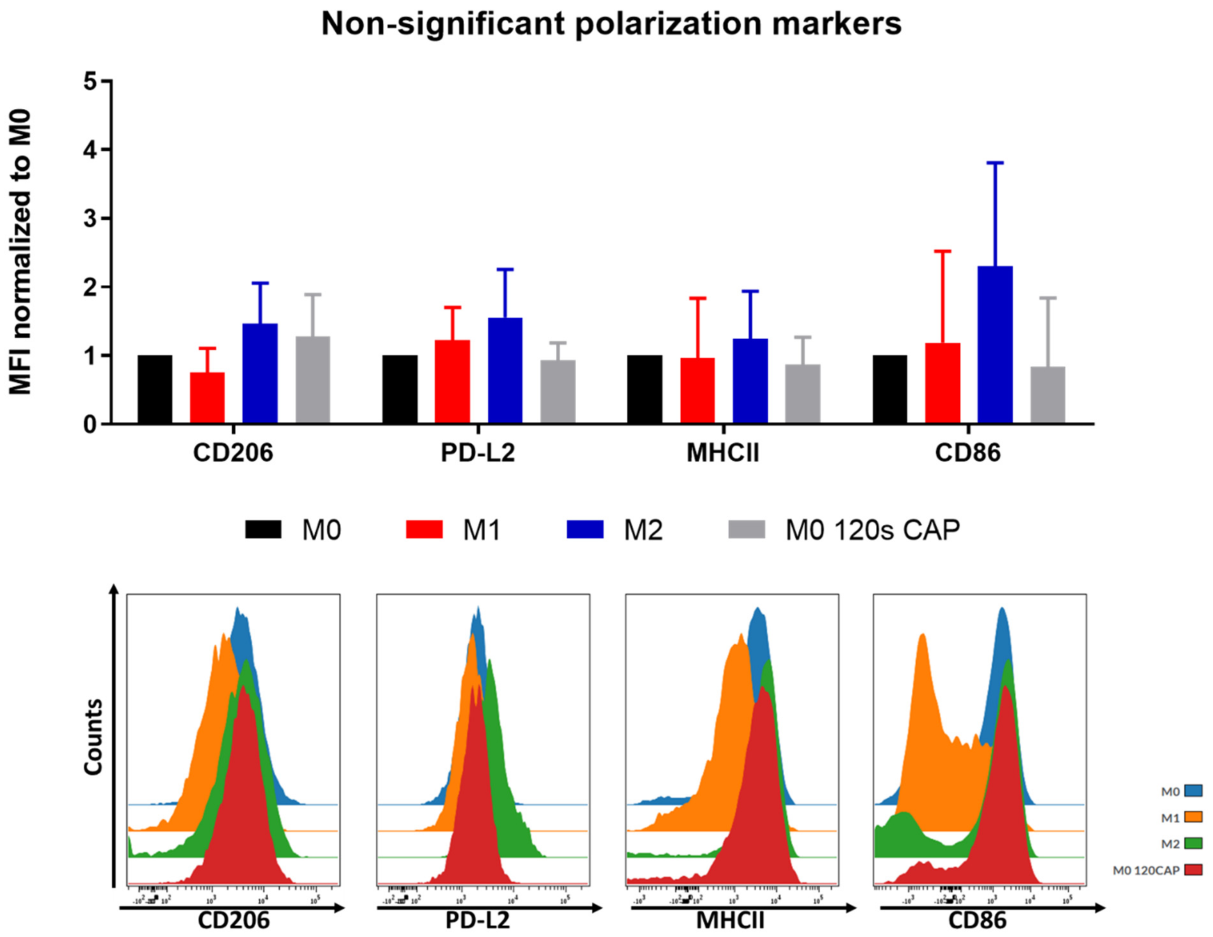

Figure S4. M0 macrophages were treated with 120s of CAP. M1 and M2 polarized macrophages served as a control. After 2 days, cells were analyzed via flow cytometry. Cells were stained with different macrophage polarization markers. Bar diagram shows the average mean fluorescent intensity (MFI) of each measured marker normalized to untreated M0 macrophages and represents the pooled results of twelve independent experiments (n=12 donors); Histograms paired to bar diagrams show one representative result. Statistical significance was calculated by performing two-way ANOVAs corrected for multiple comparisons with Tukey tests.

Figure S5: Example Seahorse OCR and ECAR data

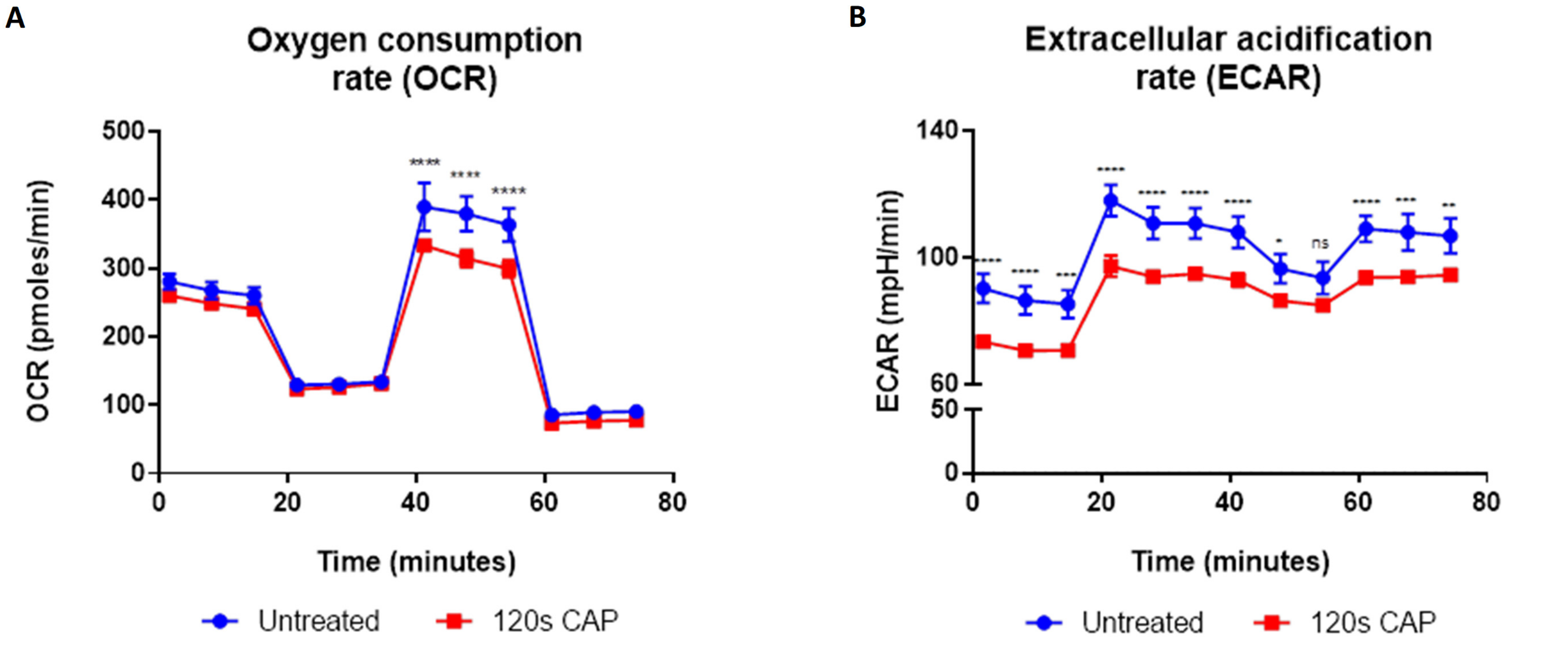

Figure S5. Representative (**A**) oxygen consumption rate (OCR) and (**B**) extracellular acidification rate (ECAR) graphs produced from a Seahorse Cell Mito Stress test are shown. Line graphs show means ± SD (n=3) of untreated and 120s CAP treated Ma-Mel-19 cells; Statistical significance was calculated by performing two-way ANOVAs corrected for multiple comparisons with Tukey tests and is indicated by the asterisks as follows: *, $P < .05$; **, $P < .01$; ***, $P < .001$; ****, $P < .0001$.

Figure S6: UKRV-Mel-15a antioxidant data

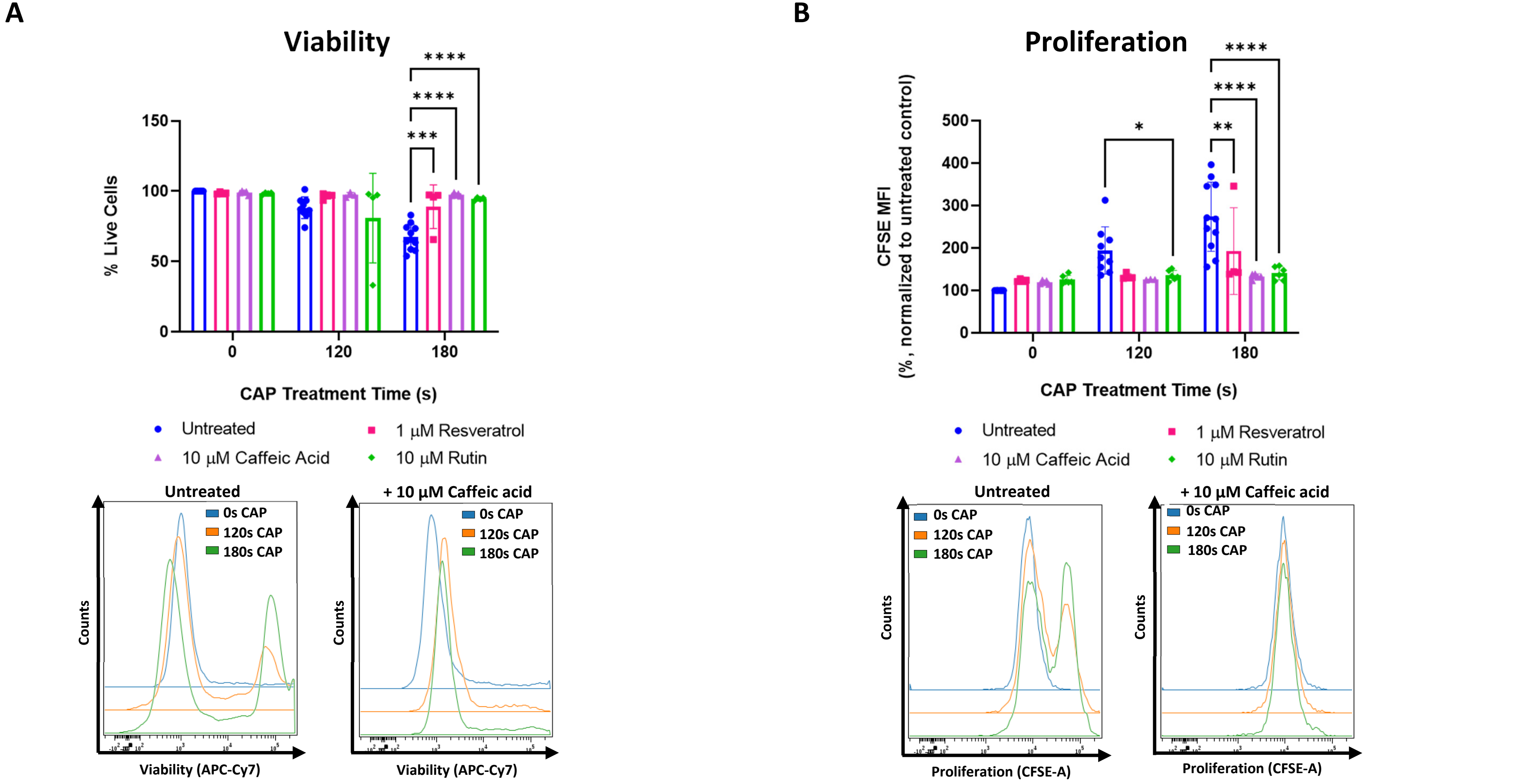

Figure S6. (**A-B**) CFSE prelabeled UKRV-Mel-15a cells were plated in the presence of various antioxidants and differentially treated with CAP (0s, 120s, 180s). After 3 days, cells were analyzed via flow cytometry; (**A**) UKRV-Mel-15a cells were stained for viability. Bar diagram shows the average percentage of live cells measured normalized to the untreated control ± SD (n=12/untreated, n=4/antioxidant); **(B**) Bar diagram shows the average CFSE MFI measured normalized to the untreated control (n=12/untreated, n=4/antioxidant) ± SD; Histograms paired to bar diagrams show one representative result. Statistical significance was calculated by performing two-way ANOVAs corrected for multiple comparisons with Dunnett tests and is indicated by the asterisks as follows: *, *P* < .05; **, *P* < .01; ***, *P* < .001; ****, *P* < .0001.

1. 10W MiniJet – HHFT. Available online: https://hhft.de/10w-minijet (accessed on 31 January 2022).
